# Functional Strength Training and Movement Performance Therapy for Upper Limb Recovery Early Poststroke—Efficacy, Neural Correlates, Predictive Markers, and Cost-Effectiveness: FAST-INdiCATE Trial

**DOI:** 10.3389/fneur.2017.00733

**Published:** 2018-01-25

**Authors:** Susan M. Hunter, Heidi Johansen-Berg, Nick Ward, Niamh C. Kennedy, Elizabeth Chandler, Christopher John Weir, John Rothwell, Alan M. Wing, Michael J. Grey, Garry Barton, Nick Malachy Leavey, Claire Havis, Roger N. Lemon, Jane Burridge, Amy Dymond, Valerie M. Pomeroy

**Affiliations:** ^1^School of Health and Rehabilitation, Institute for Applied Clinical Sciences, Keele University, Keele, United Kingdom; ^2^Wellcome Centre for Integrative Neuroimaging, Functional MRI of the Brain (FMRIB), University of Oxford, Nuffield Department of Clinical neurosciences, John Radcliffe Hospital, Oxford, United Kingdom; ^3^Sobell Department of Motor Neuroscience and Movement Disorders, UCL Institute of Neurology, London, United Kingdom; ^4^School of Psychology, Ulster University, Coleraine, United Kingdom; ^5^Faculty of Medicine and Health Sciences, University of East Anglia, Norwich Research Park, Norwich, United Kingdom; ^6^Edinburgh Clinical Trials Unit, Usher Institute of Population Health Sciences and Informatics, University of Edinburgh, Edinburgh, United Kingdom; ^7^School of Psychology, University of Birmingham, Birmingham, United Kingdom; ^8^Faculty of Health Sciences, University of Southampton, Southampton, United Kingdom

**Keywords:** stroke, rehabilitation, physical therapy, transcranial magnetic stimulation, magnetic resonance imaging, upper limb; prediction

## Abstract

**Background:**

Variation in physiological deficits underlying upper limb paresis after stroke could influence how people recover and to which physical therapy they best respond.

**Objectives:**

To determine whether functional strength training (FST) improves upper limb recovery more than movement performance therapy (MPT). To identify: (a) neural correlates of response and (b) whether pre-intervention neural characteristics predict response.

**Design:**

Explanatory investigations within a randomised, controlled, observer-blind, and multicentre trial. Randomisation was computer-generated and concealed by an independent facility until baseline measures were completed. Primary time point was outcome, after the 6-week intervention phase. Follow-up was at 6 months after stroke.

**Participants:**

With some voluntary muscle contraction in the paretic upper limb, not full dexterity, when recruited up to 60 days after an anterior cerebral circulation territory stroke.

**Interventions:**

Conventional physical therapy (CPT) plus either MPT or FST for up to 90 min-a-day, 5 days-a-week for 6 weeks. FST was “hands-off” progressive resistive exercise cemented into functional task training. MPT was “hands-on” sensory/facilitation techniques for smooth and accurate movement.

**Outcomes:**

The primary efficacy measure was the Action Research Arm Test (ARAT). Neural measures: fractional anisotropy (FA) corpus callosum midline; asymmetry of corticospinal tracts FA; and resting motor threshold (RMT) of motor-evoked potentials.

**Analysis:**

Covariance models tested ARAT change from baseline. At outcome: correlation coefficients assessed relationship between change in ARAT and neural measures; an interaction term assessed whether baseline neural characteristics predicted response.

**Results:**

288 Participants had: mean age of 72.2 (SD 12.5) years and mean ARAT 25.5 (18.2). For 240 participants with ARAT at baseline and outcome the mean change was 9.70 (11.72) for FST + CPT and 7.90 (9.18) for MPT + CPT, which did not differ statistically (*p* = 0.298). Correlations between ARAT change scores and baseline neural values were between 0.199, *p* = 0.320 for MPT + CPT RMT (*n* = 27) and −0.147, *p* = 0.385 for asymmetry of corticospinal tracts FA (*n* = 37). Interaction effects between neural values and ARAT change between baseline and outcome were not statistically significant.

**Conclusions:**

There was no significant difference in upper limb improvement between FST and MPT. Baseline neural measures did not correlate with upper limb recovery or predict therapy response.

**Trial registration:**

Current Controlled Trials: ISRCT 19090862, http://www.controlled-trials.com

## Introduction

Upper limb recovery after stroke has been identified as a top 10 research priority by people with stroke (1). This is not surprising because approximately 77% of people with stroke experience upper limb dysfunction (2), which impacts adversely on independence with everyday tasks such as drinking from a cup and fastening buttons (3). Despite benefits from physical therapy after stroke (4) for those people with initial severe paresis only around 40% will achieve some dextrous hand function at 6 months after the ictus (5, 6). Specifically; there is evidence for benefit from interventions based on repetitive task-specific training (7); the first 3 months after stroke could be the most crucial for rehabilitation (8); and there is greatest potential for neural reorganisation (neuroplasticity) (9). However, there is an increasing understanding that not everybody responds in the same way to specific physical therapies. For example, constraint-induced movement therapy is probably only suitable for people 3–9 months after stroke (10, 11) who are able to produce voluntary extension of at least 10° in the contralesional wrist and two fingers (12, 13). Essentially, there is variation in the physiological deficits resulting from stroke, which may influence how people recover and to which intervention they best respond (14). Therefore, progress requires greater understanding of the neurophysiology of therapy-induced response and which people are likely to benefit from which physical therapy (9, 14–21).

Greater understanding of the neurophysiological mechanisms of therapy-induced response is expected to inform the development of physical therapies for driving beneficial neuroplasticity (15, 19, 21). A particular benefit is expected to be provision of targets for physical therapy. For instance: focussing measurement of response on non-primary cortical motor areas, including premotor and supplementary motor area (19, 22) especially when there is greater damage to the corticospinal system (23, 24). Scientific advances are also expected from a greater understanding of how the characteristics of an individual influence their therapy-induced response (15, 20, 21). More specifically, by providing predictive markers of response to different physical therapies to enable stratification of therapy to stroke survivors based on their probability of response. Such an advance would enable clinical trials of specific physical therapies in those most likely to respond, thus reducing estimated sample sizes and avoid inclusion of those who are unlikely to benefit (15, 25). There are already experimental indications that the pre-intervention degree of injury to descending white matter pathways (26, 27) and primary motor cortex activity during movement (28) could be associated with therapy-induced recovery. Needed now are adequately powered trials which employ objective sensitive neural measures to improve understanding of how specific physical therapies enhance recovery (mechanisms for targeted therapies) and which people are most likely to respond (predictive markers of therapy-induced response) (14–16, 18, 19, 29). This approach has been used successfully to investigate language recovery (30) and is expected to add specific information to evidence-based algorithms to assist therapists to prescribe physical therapy for people with stroke (31).

The trial reported here investigated the predictors of response to and mechanisms of response to specific physical therapies in subgroups of people early after stroke. It also addressed the lack of investigation of the cost-effectiveness of physical therapy interventions (21). The two well-characterised physical therapies providing the context for investigation are used in conventional physical therapy (CPT): functional strength training (FST) focussed on improving the ability to perform everyday tasks (32–41); and movement performance therapy (MPT) focussed on improving the quality of movement during performance of everyday tasks (32, 39–43), referred to as extra CPT in early phase studies (32, 39–41). A recent systematic review concluded that such conceptually different physical therapies are probably no less or more effective than each other (4) although early phase trials indicate variation between people in their response to FST and MPT (32, 41, 44, 45).

The main aims for the trial reported here were as follows:

1. to determine whether there is greater clinical efficacy for FST that for MPT when given in addition to CPT early after stroke (FST + CPT and MPT + CPT);
2. to explore similarities and differences in neural correlates of clinical improvement in response to FST + CPT and MPT + CPT;
3. to explore the relationship between baseline measurements and improvement in upper limb motor function in response to FST + CPT and MPT + CPT.
  In addition, we aimed:
4. to provide estimates of cost-effectiveness of FST + CPT versus MPT + CPT in preparation for the development of any subsequent studies.

## Materials and Methods

The detailed trial protocol has been published (46) and is also available online (excluding the health economics component) in the report to funder (47). Consequently, the key methodological features have been previously reported and are therefore only summarised below. The trial was registered on Current Controlled Trial^1^ (ISRCTN 19090862) and adopted by the UK Clinical Research Network.

### Design, Randomisation, and Ethics

A randomised, controlled, observer-blind, two-group, and multicentre trial. Baseline measures were made for each participant before random allocation to a group. Outcome measures were undertaken on the working day (±7 days) after the 6-week intervention phase ended. Follow-up measures were undertaken six calendar months (±14 days) after the date of the index stroke. Clinical efficacy measures were made at each time point. Neural measures were made at baseline and outcome. For health economics data, EQ 5-D data were collected at all three time points and resource use at follow-up.

An independently pre-generated randomisation sequence allocated participants into two groups stratified by: clinical centre (Stoke-on-Trent, Birmingham, Norwich); time (up to 30 days or 31+ days) poststroke at time of providing informed consent: and substantial or moderate impairment in the contralesional (paretic) upper limb assessed by an inability to complete the Nine Hole Peg Test in 50 s (48) (substantial impairment = able to move one peg or less in 50 s, moderate impairment = able to move two or more pegs in 50 s.). Allocation concealment was maintained before computer-generated randomisation that was revealed by an independent telephone service for each participant after baseline clinical efficacy measures were completed.

At baseline (pre-randomisation) all assessors were blinded to group allocation. At outcome and follow-up, there were a few occurrences when a blinded assessor was unavailable. Every effort was made to ensure that all randomised participants undertook outcome and follow-up measures. When participants could not be included in an assessment the reason was documented.

Ethical approval was granted by the Norfolk Research Ethics Service (reference number 11/EE/0524).

### Participants and Screening

Adults aged 18+ years, after anterior circulation ischaemic or haemorrhagic stroke (confirmed by clinical neuroimaging) within the previous 2–60 days; scoring >11/33 on Motricity Index pinch subsection (49) with more paretic (contralesional) upper limb; unable to complete Nine-Hole Peg Test (48) in 50 s or less with paretic upper limb; no obvious spatial neglect [score of 0 or 1 on NIHSS Extinction and Inattention subsection (50)], no obvious dyspraxia or communication deficits (ability to imitate action with non-paretic limb following instruction); and able to lift a cup with paretic upper limb to take a drink before the index stroke.

Screening was undertaken in two stages. Stage 1 was undertaken by research nurses employed through UK National Institute of Health Research funding. The research nurses screened for: age; time after stroke; that the stroke was in the territory of the anterior circulation; that medical stability/fitness to participate in therapy had been reached; and that the person had been able to use their more paretic upper limb before the index stroke. Informed consent was taken by either the research nurses or a clinically qualified member of the research team. Stage 2 screening for the remaining cognitive and motor criteria was undertaken by a clinically qualified member of the research team.

### Sample Size

The sample size calculation was based on Action Research Arm Test (ARAT) (51) data from the early phase trial of FST + CPT and MPT + CPT for the upper limb (32). The power calculation accounted for the three centres and clustering in the design (participants, within a therapist within a group) and allowed for a 10% attrition rate. This provided an estimated sample size of 288 (144 participants per group) with 80% power to detect a clinically important mean difference (6.2 points on the ARAT), applying a 5% two-sided significance level.

### Interventions

The interventions have been described in detail elsewhere (47, 52–54). Consequently, only the essential elements pertinent to the present trial are here. The actual dose and content of therapy received by participants are detailed in the results section of this report.

All participants continued to receive routine CPT throughout the trial provided by the usual clinical team. The content and dose (amount in minutes) of CPT was recorded on a standardised form (52) using the same procedure as in earlier trials (32, 39–41, 55–57). A CPT-only group was not employed in this trial since additional therapy is known to be beneficial (21, 58), and the routine dose of CPT may be suboptimal (32, 41, 55, 57, 59–61).

In addition to routine CPT, participants received their experimental intervention, either FST (FST + CPT) or MPT (MPT + CPT) according to their randomised group allocation. The clinical team was not told the group allocation of participants.

Experimental interventions were provided for up to 90 min/day, 5 days a week (Monday–Friday), for a period of up to 6 weeks (6-week intervention phase). Intervention sessions were provided where participants were located: in-patient or out-patient facility, or in their own home. Therapy content and dose were recorded by the research therapists for each day on which people participated in their allocated intervention.

Experimental FST and MPT was prescribed and delivered, through direct and non-direct contact, by research therapists in each centre who were trained in wither FST or MPT according to a standardised manual. Training was provided before the first participant was randomised: any research therapist subsequently joining the trial after this point was provided with bespoke training before their first participant was randomised. During the course of the trial two group-training events were held, which provided refresher training, and enhanced the within-trial regular research therapist networking that was an important element of maintaining adherence to protocol across centres. Treatment fidelity was observed at each site on an *ad hoc* basis by a senior member of the research team.

#### Conventional Physical Therapy

Conventional physical therapy for stroke is a complex intervention (62) that combines different treatment approaches and techniques to provide personalised treatment based on comprehensive patient assessment. In the UK, CPT for the paretic upper limb includes the intervention categories: hands-on therapy to mobilise joints/soft tissues; hands-on therapy and cueing to facilitate muscle activity and movement; positioning and splinting to promote normal alignment of body structures; somatosensory stimulation; strengthening exercise; functional activities; and education (52). CPT therefore includes both MPT and FST.

The strength training elements of CPT include strengthening exercise and functional activity retraining based on findings that upper limb muscle strength is associated with ability to perform activities of daily living (33–35, 63). However, in CPT, unlike the experimental FST (see Functional Strength Training), progression of strengthening exercises and functional task training is not systematic. Instead, functional activity is encouraged as voluntary activation improves but attention to movement performance parameters remains a focus. Progression of activity is based on clinical assessment of movement performance in relation to these parameters.

The hands-on and sensory stimulation components of CPT, that focus on restoring movement quality, i.e., efficient, smooth, timely, coordinated movement with normal alignment or symmetry of body structures before retraining functional movement, is called MPT (39). The MPT elements of CPT consist of the hands-on therapy focussed on joint/soft tissue mobilisation, facilitation of muscle activity, and movement and somatosensory stimulation.

#### Movement Performance Therapy

Movement performance therapy is “therapist dependent,” particularly in the presence of limited voluntary muscle activation. The research therapists monitored and provided intrinsic feedback on movement performance through skilled observation, handling and facilitation techniques. Therapist-led hands-on guidance and feedback assisted practice of functional tasks, sometimes with increasing resistance or mass of an object and changes in speed of movement to increase the challenge of the activity, but this was not progressed systematically. Replicated single system studies of one module of MPT, known as mobilisation and tactile stimulation delivered daily for up to 60 min/day, 5 days/week for 6 weeks to the paretic upper limb of people in the subacute (54) and chronic phases after stroke (45) produced improvements in upper limb muscle strength and activity capacity.

#### Functional Strength Training

Functional strength training for the upper limb after stroke combines strength training of weaker muscles with task-specific training through “repetitive, progressive, resistance exercise during goal-directed functional activity” (53). Research therapists provided external feedback and verbal prompting whilst only using hands-on contact for safety (“therapist-independent”). The focus was on achieving key components of upper limb function (reach and grasp) through systematic progression of resisted strengthening of muscles that lacked appropriate force during goal-directed functional activity. For example, progressive strengthening of muscles around the shoulder and elbow enables more appropriate placement of the hand during reach; and improving appropriate muscle force production in the forearm, wrist, and hand improves grasp and ability to manipulate objects (53). Thus, strength gains in one component of a task are transferred to improvements in the whole task (64). Variations in types of muscle contraction (isometric, isotonic, and isokinetic) (65), number of repetitions, speed, or range of movement/distance moved, resistance (gravity, weights, and resistance bands), or changes to mass and shape of everyday objects used in the tasks, progresses the activities systematically (53). Using principles of repetitive task-specific training to relearn skilled movement and tasks, components of complex functional tasks are practiced (part practice) until the whole task can be completed actively and practiced (whole practice) with sufficiently high number of repetitions for motor learning (66) and cortical reorganisation (9).

#### Processing of Therapy Data

Dose of physical therapy is thought to be especially important as it has been associated with enhanced outcome [e.g., Ref. (21, 58)]. However, there are systematic review findings that different forms of physical therapy may confound any association (67). Therefore, the therapy data, amount (dose) and content, were extracted and collated for each randomised participant from the standardised forms completed by the clinical (CPT) and research (FST and MPT) therapists. The total hours provided for each participant of CPT and their allocated experimental intervention (FST or MPT), total number of sessions of CPT and experimental therapy provided for each participant, the mean duration (minutes) of each therapy session for each participant, and the percentage of sessions in which the categories of CPT and experimental intervention were provided, were calculated for each participant using Microsoft^®^ Excel^®^. In addition, the percentage of experimental sessions lasting 90 min or more was calculated. The reasons for a duration of less than 90 min were collated as a percentage of experimental sessions delivered.

### Outcomes—Clinical Efficacy

The time points for measures were before randomisation (baseline), the working day (±7 days) after the 6-week intervention phase ended (outcome) and six calendar months (±14 days) after the date of the index stroke (follow-up). Outcome was the primary endpoint.

#### Primary Outcome Measure

The primary outcome measure was the ARAT as a measure of the expected effect of both experimental interventions: recovery of upper limb activity capacity (51).

#### Secondary Outcome Measures

The Wolf Motor Function Test (WMFT) as both an extended measure of upper limb activity capacity and the quality of movement during the 15 test tasks (68, 69). Other measures were hand grip and pinch grip forces, direct measures of muscle strength using a myometer held securely in a purpose-built device placed on a stable surface and using a standardised position for upper limb position (70, 71).

### Outcomes—Neural

This report focuses on the prespecified neural outcomes, listed below, which were chosen to be congruent with recommendations for choice of biomarkers of motor recovery after stroke (25). Subsequent reports will present exploratory analyses of other measures derived from the neuroimaging data.

#### Neuroimaging

Neuroimaging was acquired on hospital or university 3T magnetic resonance imaging (MRI) scanners at each research site. These included a Siemens Skyra (Stoke-on-Trent), Philips 3T Achieva (University of Birmingham), and GE Discovery MR750w (Norwich). Effort was made to match neuroimaging protocols across sites as far as possible, thought slight variation in scan sequences was unavoidable due to scanner differences. Scans included the following:

- A T_1_-weighted 1 mm × 1 mm × 1 mm, whole-brain image was obtained using structural MRI [Norwich (GE): BRAVO: TI/TE/TR = 900/6/14.1 ms, FOV = 25.6 cm, acquisition matrix = 256 × 256; Birmingham (Philips): 3DTFE: TI/TE/TR = 900/4.7/11.2 ms, FOV = 25.6 cm, acquisition matrix = 256 × 256; Stoke-on-Trent (Siemens): Skyra: TI/TE/TR = 900/3.51/2,040 ms, FOV = 256 × 192, acquisition matrix = 256 × 256].
- Diffusion tensor imaging (DTI) scans were acquired at 2 mm × 2 mm × 2 mm (64 isotropically distributed diffusion directions; *b* = 1,500; 62 slices; plus two sets of *b* = 0 images, one with phase encoding anterior to posterior, and the other with phase encoding posterior to anterior).
- Dual-echoT_2_-weighted and proton density whole brain MRI scans (GE: TE = 13 ms; TE2 = 101 ms; and TR = 4,670 ms) were acquired at 1 mm × 1 mm × 3 mm to enable accurate delineation of stroke volumes.

Task-based and resting state functional MRI data were also acquired when possible but are not reported here due to small subject numbers.

Each neuroimaging scan was sent promptly to the Oxford-based team members to undertake quality control checks. Quality control assessments included manual checks (e.g., subject motion) and automated checks (e.g., signal to noise, motion correction parameters, and range checks). Those scans meeting the required quality standard were processed using tools from FMRIB’s Software Library (FSL^2^). The lesion was delineated manually on each patient’s T1-weighted 1 mm × 1 mm × 1 mm brain image using FSL view, an image viewing tool available within FSL.

Measures extracted were as follows:

- The anatomical overlap of the stroke lesion with the Montreal Neurological Institute corticospinal tract (MNI CST)—yes/no: this variable identifies whether the lesion overlaps with a mask approximating the corticospinal tract. The CST mask was defined using tractography in a group of control brains in standard space to track between motor cortex and medullary pyramids. Overlap between the lesion volume and the CST was determined by overlaying the lesion mask, described earlier, with the CST mask.
- The volume of the stroke lesion (lesion volume, mm^3^): this variable was determined by calculating the volume of the manually defined stroke mask described earlier.
- Corticocortical anatomical connectivity as per fractional aniostrophy from corpus callosum midline [fractional anisotropy (FA) MNI corpus callosum misline—range = 0–1]: DTI data were first corrected for head motion and eddy current using tools from FSL. A diffusion tensor model was then fitted to the preprocessed data to calculate voxel-wise values of FA. Mean FA from within a standard space region of interest, incorporating the whole corpus callosum on a midline brain slice, was calculated.
- Corticospinal anatomical integrity as per asymmetry of corticospinal tract FA (ipsilesional:contralesional MNI CST—range = −1 to 1): regions of interest on a single slice at the level of the internal capsule were created to provide masks estimating the location of the CST. Mean FA from within these masks was calculated. A ratio of ipsilesional:contralesional values was calculated.

#### Transcranial Magnetic Stimulation (TMS)

Single pulse TMS was applied over the hand/arm area of the ipsilesional primary motor cortex (M1) to elicit a motor-evoked potential (MEP). The coil was oriented at approximately 45° to the sagittal plane, to induce posterior to anterior current flow across the motor strip in the primary motor cortex. Stimuli were delivered with a Magstim 200^2^ stimulator (The Magstim Company Ltd., UK) *via* a standard 70 mm figure-of-eight coil with intensities between 100 and 130% of active motor threshold. MEPs were detected in the electromyogram *via* skin surface electrodes placed over the more paretic extensor carpi radialis (pECR) and paretic biceps brachii (pBB) muscles. All EMG records were amplified (500–2 k), band pass filtered (20–1,000 Hz), and digitally sampled at 5 kHz (Digitimer Neurolog System) to be stored for offline analysis (Signal, CED). The resting motor threshold (RMT) was determined as the intensity at which at least 5 out of 10 stimuli evoked MEPs with a peak-to-peak amplitude of greater than 50 µV (72). Active motor threshold was determined during a slight contraction of the target muscles (BB and ECR) at approximately 20% of the maximal muscle strength (72). Measures extracted were focussed around threshold MEP measures as these are probably the most reliable (73):

- corticospinal functional connectivity as per presence or absence of a resting MEP for pBB (pMEPBB—yes/no);
- corticospinal functional connectivity as per presence or absence of a resting MEP for pECR [pMEPECR (yes/no)];
- RMT in pBB when MEP was present [RMTpBB (%)];
- RMT in pECR when MEP was present [RMTpEXR (% maximum stimulator output)].

A number of key measurements including TMS threshold, stimulation intensity, presence, or absence of an MEP were recorded on the trial TMS case report (CRF) form for each individual and each session. These data were collated by the Clinical Trial Unit (CTU) to provide details on the number of participants who displayed an MEP in target muscles (ECR and BB) in both limbs at resting and active motor threshold.

### Outcomes—Health Economics

#### Intervention Costs

We estimated the total NHS and Personal Social Services (PSS) cost, without separately estimating the cost of the therapy intervention visits. It was assumed that no additional training or equipment costs would be required to roll the MPT out into practice. If FST was to be rolled into practice some centres may not have the equipment used in this trial, e.g.: resistive exercise bands, therapy putty, foam tubing (to help hold pens/cutlery), and graded pinch pins. A log of all equipment purchases was kept. A log was kept for all training delivered, including workshop times for the trainer(s) (including preparation) and trainee(s), and distances travelled, accommodation, and room hire costs. The trainers were cost as Band 8a staff [£62 per hour of employment (74)], and those delivering therapy were cost as Band 6 staff [£44 per employment hour (74)]. The total costs associated with both equipment and training were estimated and equally apportioned across all participants.

#### Other NHS and PSS Costs

At follow-up, participants were asked to provide details of the use of all health-care services (including social care) since the index stroke, i.e., last 6 months. Unit costs (2014/2015 financial year levels) were assigned to all reported items of resource (74–77). This enabled the total NHS and PSS costs to be estimated. No discounting was undertaken as the follow-up period was less than 1 year.

For each health-care service, a Yes, No, or participant cannot remember option was available. Where a No was reported the costs for that item of resource were assumed to be 0 and if the participant reported that they could not remember, the data were assumed to be missing. If they answered Yes, they were asked to report details including the level of resource use for that item in the previous 6 months.

Participants were asked if they had consulted or been visited by a health-care professional. We assumed that participants included therapy intervention visits within reported data. Unit costs [e.g., Ref. (74, 76)] were assigned to all visits.

Participants were asked to report if they had taken any medication (prescribed by the doctor). Those who responded yes, were asked to report the name of the medication, along with the associated dosage and whether they were taking the drug on an ongoing basis. Unit costs were subsequently attached to each reported medication based on the net ingredient cost per item for the individual preparation (75). Where the medication dosage was unclear, the weighted average net ingredient cost per item for that medication was used, where the medication box was completed but that could not be matched to an individual preparation, the mean net ingredient cost per item for all items was used. It was assumed that prescriptions reported as ongoing at the follow-up point had been taken for the whole of the prior 6 months, whereas those that were not reported as ongoing had only been taken once during the previous 6 months.

Participants were asked if they had been admitted to hospital. Those that responded with “yes” were asked to provide the admission and discharge date for each occasion. A unit cost per bed day (77) was assigned to each day of an admission.

Participants were asked if they had attended accident and emergency department (A&E). A unit cost (77) was assigned to each visit.

Participants were asked if they had stayed in another place of residence other than their own home (not including hospital admissions). Unit costs (74) were then assigned.

Finally participants were asked if they had received any further help/care (not reported previously). In the base-case analysis, unit costs (74) were only assigned to care that was reported to be provided by a professional carer but was not paid for by the participant.

#### Quality of Life

In line with the National Institute for Health and Clinical Excellence (NICE) methods guide (78), quality of life was measured using the EQ-5D-3L (79). Responses were converted into utility scores (80) using the York A1 tariff (81). Quality-Adjusted Life Year (QALY) scores were subsequently calculated at follow-up using the total area under the curve approach (82) for all those who completed the EQ-5D-3L at baseline and follow-up.

### Adverse Reactions and Events

The potential adverse reactions to participation in the exercise-based therapies evaluated in this trial were considered to be pain and fatigue. Pain was held to be an adverse reaction if: (a) a participant expressed onset of pain in the paretic upper limb (verbally or behaviourally); (b) this was sustained over four consecutive therapy occasions; and (c) the clinical team had no explanation of pain onset other than involvement in the trial reported here. Fatigue was held to be an adverse reaction if: (a) a participant had a decrease of two levels of the upper limb Motricity Index on two consecutive therapy occasions and (b) the clinical team could not attribute fatigue to anything other than involvement in the trial reported here. If either adverse reaction occurred the trial therapy was adjusted or stopped, whichever was most appropriate for an individual.

Adverse events were recorded in accordance with a standard operating procedure (SOP 25: latest version at http://www.nnuh.nhs.uk/publication/sop-205-adverse-events/).

### Statistical Analysis

Before the database lock and unblinding of group allocations, the statistical analysis plan (SAP) was agreed, signed, and dated. In the agreed SAP, the analysis plan adjusted for study site which was a change from original intention of also accounting for clustering of patients by therapist. This change was made because therapist changes during the course of the trial meant that participants did not receive treatment by the same therapist for across all of their experimental intervention sessions. The SAP did not include the health economics analysis or statistical analysis of therapy data, though some detail of the associated planned analysis was given in the published protocol (46).

#### Clinical Efficacy (Aim 1)

Change from baseline to outcome and follow-up in the clinical efficacy variables was analysed with analysis of covariance models. These were adjusted for the baseline values and randomisation strata (time after stroke, ability to use contralesional upper limb and clinical centre). A log or other appropriate transformation was applied where the outcome/follow-up distribution deviated from a normal distribution. Adjusted least squares mean difference and associated 95% confidence intervals (95% CIs) were calculated. The association (Pearson correlation coefficient) between the total dose of CPT plus either FST or MPT was scrutinised to check for influence on response to therapy dose received.

#### Neural Correlates of Clinical Improvement (Aim 2)

Associations between the changes from baseline to outcome of the neural variables and the clinical efficacy variables were assessed. The correlation coefficient was calculated for the two intervention groups separately and for combination of the groups.

#### Predictive Neural Markers of Clinical Improvement (Aim 3)

For each baseline covariate investigated as a potential predictive marker of clinical improvement, the treatment effect was calculated within each level of the subgroup (adjusted as in the clinical efficacy analysis). An interaction term between randomised treatment and the baseline covariate was included in the model.

#### Estimates of Cost-Effectiveness (Aim 4)

In the base-case analysis a complete case approach (83) was undertaken, whereby participants were only included if the overall estimated cost to the NHS and PSS and the QALY score could be calculated.

For those participants with complete data, bivariate regression (84) was used to estimate the mean incremental cost between the two groups (mean difference in overall cost to the NHS and PSS) and the mean incremental effect (mean per participant difference in QALYs). Cost and effect regression analyses were run simultaneously, with age and sex included as covariates, plus the baseline EQ-5D score for the QALY regression.

The mean per participant incremental cost and incremental effect were subsequently used to estimate the incremental cost-effectiveness ratio (ICER) (80). In the UK, NICE refers to a cost-effectiveness threshold (λ) value of £20,000–30,000 per QALY (78), and we considered that an estimated ICER below £20,000 would indicate that the intervention constituted value for money. However, due to the potential for a negative ICER to be estimated (which could result from either a negative cost or negative effect), as there is potential for this to be misinterpreted (85), the net benefit was also calculated at λ = £20,000 per QALY. A positive value indicates that the intervention was estimated to be cost-effective at that threshold (85).

To estimate the level of uncertainty associated with the decision regarding cost-effectiveness, bootstrap resampling (86) (with 5,000 replications) was used to estimate the cost-effectiveness acceptability curve (CEAC) (87). The CEAC depicts the probability of the intervention being cost-effective at various “willingness to pay” thresholds compared to standard care. Here, the probability was estimated at a λ (threshold) value of £20,000 per QALY.

Sensitivity analyses (80) were undertaken to assess the robustness of conclusions to changes in key assumptions. Within the first sensitivity analysis (SA1), overall NHS and PSS costs and QALYs were rerun following multiple imputation (88) to account for missing cost and outcome data. We performed multiple imputation in a single model using the mi impute command in Stata 14. In addition to the costs and EQ-5D scores, the MI model included age, sex, and treatment allocation. Imputation took place in twenty cycles, the estimates from which were then pooled and calculated using Rubin’s rules (89). In a further sensitivity analysis (SA2), the base-case analysis was replicated but we excluded the intervention costs relating to training and equipment, on the assumption that these costs would not need to be incurred again if the intervention continued. The third sensitivity analysis (SA3) additionally included professional carer costs if they were reported to be paid for by the participant. A fourth sensitivity analysis (SA4) included these professional carer costs as well as any other care that the participant reported receiving, where the reported hours of care were assigned the mean hourly pay for all UK employees (90). A fifth sensitivity analysis (SA5) was undertaken to assess whether results were particularly sensitive to outliers using data for participants whose estimated overall cost to the NHS and PSS was above the 95th percentile and below the 5th percentile was excluded from the analysis.

### Trial Management

Trial data were collected in accordance with trial operating procedures, developed before the first participant was randomised, to the standard required in the SOPs agreed for use by the University of East Anglia (trial sponsor). The completed case report forms (CRF) were secured in a locked filing cabinet in research offices at each centre. When the trial follow-up phase was complete, all CRFs were transferred securely to the University of East Anglia where the final data query resolution was undertaken before database hardlock. All CRFs are stored securely at the University of East Anglia where they will be archived.

The data management team at the Glasgow CTU set up and managed the trial database in accordance with a data management plan. This included procedures for maintaining blinding, and limited access of members of the trial team to data throughout the trial. For example, data were sent to the Glasgow CTU in a secure standardised manner for database entry and quality control checks. An SAP for the clinical efficacy and explanatory data was agreed between the Glasgow CTU and research team before database lock and unblinding of group allocation.

The therapy data (content and dose of CPT, FST, and MPT) were not considered essential to answer the trial aims by the Glasgow CTU. Consequently, the trial sponsor (UEA) agreed an independent quality control review of: (a) the therapy data in the raw database supplied by the Glasgow CTU and (b) the processing undertaken by the research team using Microsoft^®^ Excel^®^ spreadsheets to provide values for statistical analysis. The values used in the statistical analysis have therefore been quality assured by an auditor independent of the research team.

A Trial Steering Committee (TSC) with independent chair and members was convened to provide overall supervision and ensure good conduct of the trial. The TSC met on nine occasions.

A Data Monitoring and Ethics Committee (DMEC), with independent chair and members appointed by the funder, reported directly to the chair of the TSC. The DMEC met six times.

## Results

### Flow of Participants through the Trial

**Figure 1:**
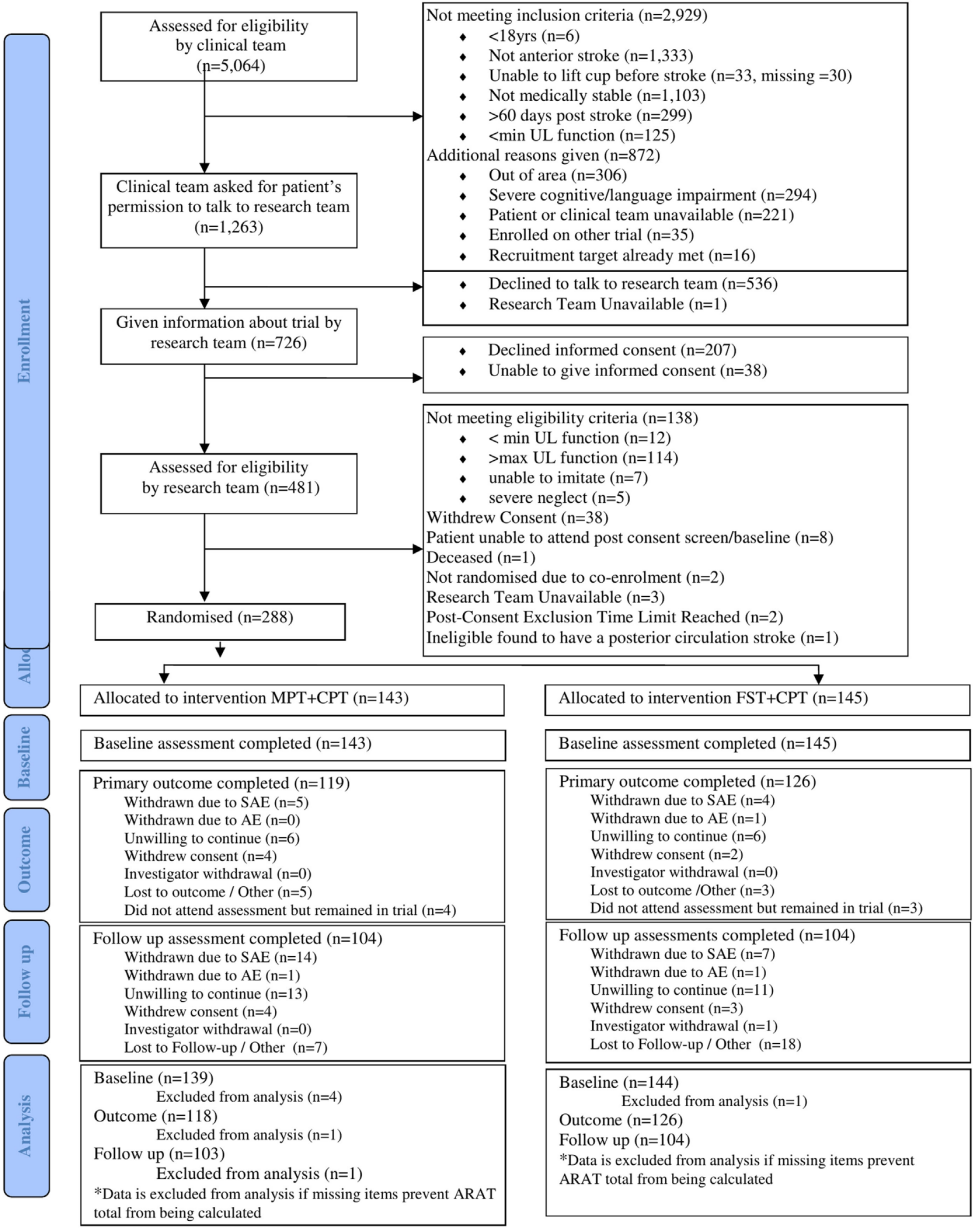
All centres: CONSORT 2010 flow diagram.

The CONSORT flowchart for this trial (Figure 1) shows that 5,064 people with stroke were screened by the research nurses (stage 1), of whom 1,263 (24.9%) were potentially eligible for this trial. However, 536 (42.4%) of those declined to talk to the research team and the research team were unavailable to talk with one potential participant. A further 207 people declined informed consent and 38 were unable to provide informed consent. The remaining 481 provided informed consent and undertook stage 2 screening with a clinically qualified research team member. Of the 481 potential participants, 138 did not meet the cognitive and motor criteria and a further 55 either withdrew informed consent or were not randomised (reasons detailed in Figure 1). Consequently, 288 (5.7%) of the 5,064 people screened were randomised into this trial; 145 were allocated to FST + CPT and 143 to MPT + CPT. The attrition rate at outcome was 12.5% and at follow-up was 27.8%.

MR scans were undertaken by those participants who had no contraindications, had provided separate informed consent for this part of the assessment, and who could travel to the neuroimaging facility. MR scans were undertaken for 94 (32.6%) of participants at baseline and 62 (23.0%) at outcome (Table 1). Reasons for non-completion of MRI recorded in the hard-locked raw database, are shown in Table 1.

**Table 1:** Magnetic resonance imaging (MRI) (neuroimaging) and transcranial magnetic stimulation (TMS) data acquisition at baseline and outcome.

	Baseline (*n* = 288^a^)		Outcome (*n* = 245^a,b^)	

	Number	%	Number	%
**MRI data**				
Attended for neuroimaging	94	32.6	62	23.0
Unable to attend, unwell or unable to contact	35	12.2	46	17.1
Did not consent for MRI	56	19.4	71	26.4
Site approval, governance, or protocol not yet in place	32	11.1	23	8.6
Deemed unsuitable for MR, e.g., unable lie flat	8	2.8	3	1.1
Contraindications to MR	44	15.3	37	13.8
Scanner not available	17	5.9	16	6.0
No baseline scan	–	–	3	1.1
Out of area, in nursing home or SAE	1	0.3	8	3.0
Out of protocol time limit	1	0.3	0	0.0

**TMS data**				
Attended for TMS	111	38.5	83	30.2
Did not consent for TMS	37	12.8	43	15.6
Unable to attend	25	8.7	44	16.0
Site approval, governance, or protocol not yet in place	12	4.2	13	4.7
Deemed unsuitable for TMS	6	2.1	0	0.0
Contraindications to TMS	69	24.0	64	23.3
Concomitant disease	1	0.3	1	0.4
TMS not available	24	8.3	16	5.8
Out of area, in nursing home or SAE	3	1.0	8	2.9
Data missing	0	0.0	3	1.1

*^a^Data obtained from Clinical Trial Unit (CTU) locked raw database*.

*^b^CTU outcome data account for: 269 participants in terms of attendance and non-attendance for MRI scanning; and 275 participants in terms of attendance and non-attendance for TMS (*n* = 245 refers to the number of participants with a total score for the Action Research Arm Test at outcome)*.

Transcranial magnetic stimulation was undertaken by those participants who had no contraindications. The TMS measures were made with 111 (38.5%) participants at baseline and 83 (30.2%) at outcome (Table 1). Reasons for no TMS as recorded in the hard-locked database are shown in Table 1.

### Participant Characteristics at Baseline

Table 2 provides details of the demographic and clinical (efficacy) characteristics of participants randomised into this trial. In summary: mean (SD) age was 72.2 (12.5) years, with 64.6% male. The anterior circulation stroke lesions were classified as hemorrhagic for 8.7% of participants and ischaemic for 91.3%. The left side of the body was more affected (paretic side) for 58.0% of participants. Of the 288 participants, 59% provided informed consent within 30 days of stroke.

**Table 2:** Participant characteristics, demographics, and clinical (efficacy), at baseline.

	Statistic	FST + CPT (*n* = 145)	MPT + CPT (*n* = 143)	All (*n* = 288)
Age (years)	Mean (SD)	71.9 (12.7)	72.4 (12.3)	72.2 (12.5)
Sex	Male (%)	96 (66.2)	90 (62.9)	186 (64.6)
	Female (%)	49 (33.8)	53 (37.1)	102 (35.4)
Type of stroke	Ischaemic (%)	131 (90.3)	132 (92.3)	263 (91.3)
	Haemorrhagic (%)	14 (9.7)	11 (7.7)	25 (8.7)
Side of brain lesion	Left (%)	63 (43.5)	58 (40.6)	121 (42.0)
	Right (%)	82 (56.5)	85 (59.4)	167 (58.0)
NHPT at consent	1 peg or less (%)	91 (62.8)	90 (63.0)	181 (62.9)
	2–8 pegs (%)	54 (37.2)	53 (37.1)	107 (37.2)
Days after stroke at consent	≤30 days (%)	86 (59.3)	84 (58.7)	170 (59.0)
	31 days+ (%)	59 (40.7)	59 (41.3)	118 (41.0)
ARAT total—contralesional	Mean (SD)	24.7 (18.9)^a^	26.2 (17.4)^a^	25.5 (18.2)^a^
WMFT—performance	Mean (SD)	36.4 (20.25)^b^	37.6 (17.1)^b^	37.0 (18.8)^b^
Grip force (kg)	Mean (SD)	7.6 (8.7)^c^	6.9 (8.1)^c^	7.2 (8.4)^c^
Pinch force (kg)	Mean (SD)	2.2 (2.2)^d^	1.9 (2.3)^d^	2.1 (2.2)^d^

*NHPT, Nine-Hole Peg Test; ARAT, Action Research Arm Test; WMFT, Wolf Motor Function Test; FST, functional strength training; CPT, conventional physical therapy; MPT, movement performance therapy*.

*^a^Number of data sets available for analysis were as follows: FST + CPT = 144; MPT + CPT = 139; and all = 283*.

*^b^Number of data sets available for analysis were as follows: FST + CPT = 136, MPT + CPT = 129; and all = 265*.

*^c^Number of data sets available for analysis were as follows: FST + CPT = 141, MPT + CPT = 139; and all = 280*.

*^d^Number of data sets available for analysis were as follows: FST + CPT = 131, MPT + CPT = 133; and all = 264*.

The neural characteristics of those participants undertaking MR scans and/or TMS are shown in Table 3. The number of participants ranged from 80 (for corticocortical connectivity and corticospinal connectivity) to 110 (for MEPs in biceps brachii and extensor carpi radialis). Values were as follows: corticocortical connectivity was mean 0.4 (SD 0.08); corticospinal connectivity was mean 0.0 (SD 0.05); and lesion volume (mm^3^) was median 4,293 (IQR 845.50, 17,160). MEPs were present in biceps brachii for 73% of the 110 measured participants; and in extensor carpi radialis for 76% of the 110 measured participants. There were no discernible differences between the FST + CPT and MPT + CPT groups at baseline.

**Table 3:** Neural values, by treatment group and overall, at baseline.

	FST + CPT	MPT + CPT	All
**Corticocortical connectivity^a^**			
Number	42	38	80
mean (SD)	0.4 (0.09)	0.4 (0.08)	0.4 (0.08)
Median (IQR)	0.4 (0.34–0.47)	0.4 (0.34–0.49)	0.4 (0.34–0.47)

**Corticospinal connectivity**			
Number	42	38	80
Mean (SD)	0.0 (0.04)	0.0 (0.05)	0.0 (0.05)
Median (IQR)	0.0 (0.01–0.06)	0.0 (−0.02 to 0.06)	0.0 (0.001–0.06)

**Lesion volume (mm^3^)**			
Number	44	40	84
Mean (SD)	23,847.89 (47,664.63)	25,476.38 (59,957.23)	24,623.36 (53,542.90)
Median (IQR)	6,537.50 (1,067.50–19,551.50)	2,486.00 (474.00–14,046.50)	4,293.00 (845.50–17,160.50)

**MEP present pBB**			
Number	53	57	110
Yes (%)	37 (69.81%)	43 (75.44%)	80 (72.73%)
RMT number	37	57	110
RMT mean (SD)	61.1 (11.49)	63.0 (14.39)	62.1 (13.08)
RMT median (IQR)	60.0 (54–66)	66.0 (53–74)	60 (54–70)
No (%)	16 (30.19%)	14 (24.56%)	30 (27.27%)

**MEP present pECR**			
Number	53	57	110
Yes (%)	39 (73.58%)	45 (78.95%)	84 (76.36%)
RMT number	39	44	83
RMT mean (SD)	52.4 (12.30)	54.5 (15.27)	53.5 (13.91)
RMT median (IQR)	50 (46–58)	52.5 (44–61)	51.0 (45–60)
No (%)	14 (26.42%)	12 (21.05%)	26 (23.64%)

*MEP, motor-evoked potential; pBB, paretic biceps brachii; RMT, resting motor threshold (% stimulator output); pECR, paretic extensor carpi radialis; FST, functional strength training; CPT, conventional physical therapy; MPT, movement performance therapy; FA, fractional anisotropy*.

*^a^FA MNI corpus callosum midline, range 0–1*.

### Dose and Content of Therapy Received by All Randomised Participants

In accordance with the intention-to-treat principle, the therapy data presented in this report are for all randomised participants not just those still remaining in the trial at outcome and/or follow-up. The FST + CPT group received a mean (SD) of 7.4 (6.7) hours of CPT during the 6-week intervention phase in 12.9 (8.9) sessions per individual. This compares to a mean (SD) of 6.7 (5.8) hours of CPT for the MPT + CPT group in 12.1 (8.7) sessions. Thus, the amount of CPT delivered to both groups was essentially no different.

The mean (SD) number of hours of experimental therapy was 14.8 (SD 9.9) for the FST + CPT group, and 19.2 (11.1) for the MPT + CPT group. In total the MPT + CPT group received 4.4 h more of experimental therapy than the FST + CPT group but the variances around means were large (Table 4). This amount of therapy was delivered in a mean (SD) of 18.0 (8.6) sessions for the FST + CPT group and 19.1 (7.9) for the MPT + CPT group (Table 4).

**Table 4:** Amount (dose) of CPT and experimental (MPT or FST) delivered and reasons for sessions lasting less than 90 min for all randomised participants (not just those with total ARAT score at outcome).

	CPT received		Experimental received	
	FST + CPT (*n* = 145)	MPT + CPT (*n* = 143)	FST + CPT (*n* = 145)	MPT + CPT (*n* = 143)
Hours of intervention	*n* = 124	*n* = 124	*n* = 145	*n* = 142
Participants with records^a^ (*n*)	(85.5%)	(86.7%)	(100%)	(99.3%)
Mean (SD)	7.4 (6.7)	6.7 (5.8)	14.8 (9.9)	19.2 (11.1)
Median (IQR)	5.7 (2.2, 11.1)	5.0 (2.1, 8.9)	13.3 (6.2, 21.5)	18.2 (10.1, 26.6)
Min–Max	0–37.9	0–23.9	0.1–40.9	0–45
Number of sessions	*n* = 126	*n* = 124	*n* = 145	*n* = 142
Participants with records (*n*)	(86.2%)	(86.7%)	(100%)	(99.3%)
Mean (SD)	12.9 (8.9)	12.1 (8.7)	18.0 (8.6)	19.1 (7.9)
Median (IQR)	12.0 (6.0, 18.3)	10.0 (5.0, 19.0)	19.0 (11.0, 25.0)	21.0 (13.8, 26.0)
Min–Max	0–43	0–33	0–42	0–31
Minutes per session	*n* = 124	*n* = 124	*n* = 145	*n* = 142
Participants with records (*n*)	(85.5%)	(86.7%)	(100%)	(99.3%)
Mean (SD)	30.6 (12.9)	30.5 (13.2)	46.2 (16.9)	57.6 (17.9)
Median (IQR)	30.2 (20.0, 40.0)	30.4 (22.0, 38.0)	45.5 (34.0, 56.3)	56.5 (44.1, 71.5)
Min–Max	0–78	0–83	4.0–88.65	0–90
Reason for less than 90 min session time	NA	NA	*n* = 144	*n* = 142
Participants with records (*n*)			(99.3%)	(99.3%)
% Sessions lasting 90+ min (median, IQR)	NA	NA	0.0 (0.0, 9.2)	8.3 (0.0, 33.3)
% Fatigued = less than 90 min (median, IQR)	NA	NA	28.1 (13.3, 42.3)	35.2 (13.3, 60.0)
% Unwell = less than 90 min (median, IQR)	NA	NA	0.0 (0.0, 3.4)	0.0 (0.0, 4.0)
% Declined = less than 90 min (median, IQR)	NA	NA	13.3 (3.3, 27.1)	7.3 (0.0, 23.3)
% Other = less than 90 min (median, IQR)	NA	NA	38.5 (20.0, 64.2)	32.8 (13.3, 60.3)

*CPT, conventional physical therapy; MPT, movement performance therapy; FST, functional strength training; IQR, interquartile range; NA, not applicable; ARAT, Action Research Arm Test*.

*^a^All randomised participants and not just those with total ARAT scores at outcome*.

The mean (SD) duration (minutes) for each CPT therapy session was 30.6 (12.9) for the FST + CPT group and 30.5 (13.2) for the MPT + CPT group (Table 4). The experimental therapy sessions lasted longer with a mean (SD) of 46.2 (16.9) and 57.6 (17.9) min for the FST + CPT and MPT + CPT groups, respectively. This difference was not statistically significant (independent samples *t*-test; *t* = 0.271, *p* = 0.633).

The median (IQR) percentage of sessions lasting at least 90 min was 0.0 (0.0, 9.2) for the FST + CPT group and 8.3 (0.0, 33.3) for the MPT + CPT group. Table 4 provides details of the reasons for sessions lasting less than 90 min recorded on the case report forms as: fatigued; unwell; declined; or other.

**Figure 2:**
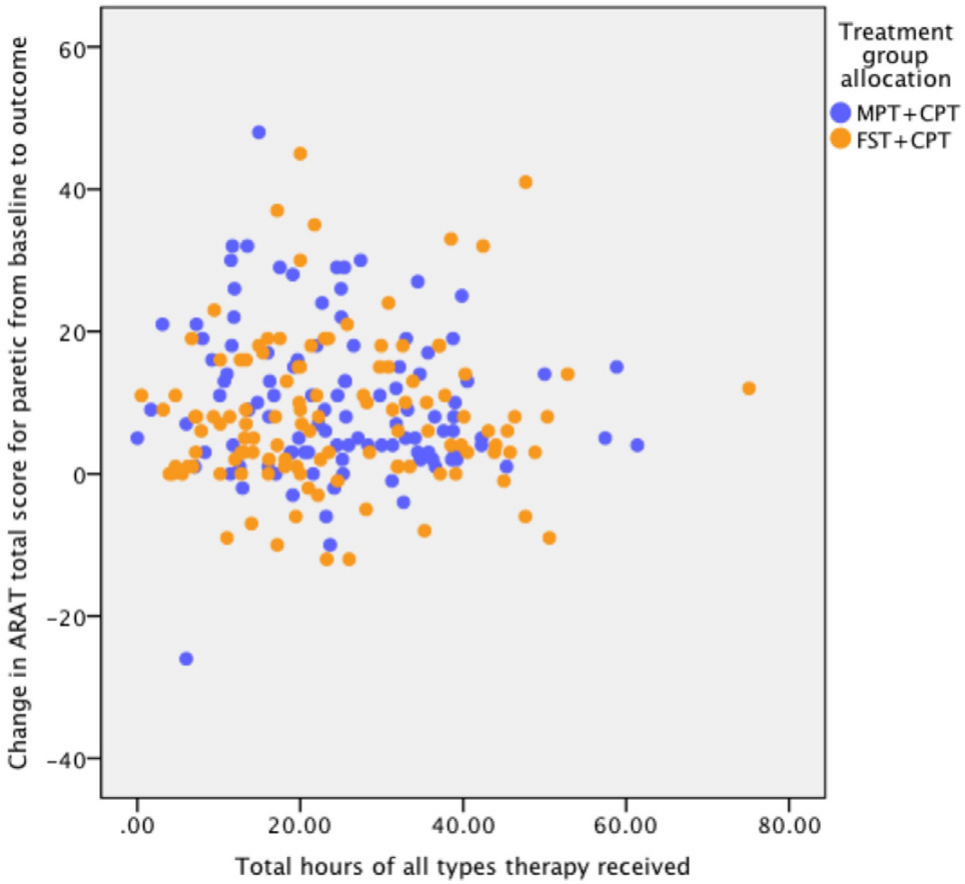
Association between dose (hours) of therapy and response to therapy as measured by change in Action Research Arm Test (ARAT) score from baseline to outcome. Pearson correlation coefficients for: FST + CPT group (n = 101) r = 0.154, p = 0.123; MPT + CPT group (n = 103) r = ?0.055, p = 0.581; and for all participants (n = 204) r = 0.071, p = 0.311.

The check for influence on response to the actual therapy dose received found no statistically significant correlation between the total hours and change in ARAT score from baseline to outcome. The results of the correlation analysis are: FST + CPT group (*n* = 101) *r* = 0.154, *p* = 0.123; MPT + CPT group (*n* = 103) *r* = −0.055, *p* = 0.581; and for all participants (*n* = 204) *r* = 0.071, *p* = 0.311 (Figure 2).

The content of the CPT delivered to participants by the clinical therapists is detailed in Table 5. In summary, the percentage of sessions in which specific modalities of CPT were provided for both groups was, essentially no different. All participants received the experimental intervention to which they had been allocated. For the FST + CPT group a median of between 73.2 and 100.0% of sessions, across four “therapist-independent” treatment categories, focussed on improving the ability to produce voluntary contraction in specific muscles required for upper limb function combined with training the ability to perform everyday functional tasks. This compares with the MPT + CPT group, where a median of: 100% of the therapist-dependent sessions included isolated joint movement and mobilisation; 75% included facilitation of muscle activity and movement; 51% included positioning; 46% included specific sensory input; and 31% included soft tissue mobilisation (Table 5). Although 93% of sessions included upper limb functional tasks and 69% included exercise to increase strength, there are differences between these FST and MPT categories not least that MPT is provided through “hands-on techniques.” These differences are delineated in the intervention protocols (47, 52–54).

**Table 5:** Treatment content for all randomised participants: percentage of sessions in which specific treatment categories were provided.

	Functional strength training (FST) + conventional physical therapy (CPT) (*n* = 145)	Movement performance therapy (MPT) + CPT (*n* = 143)

	Percentage sessions, median (IQR)	Percentage sessions, median (IQR)
**CPT**		
Number participants with treatment records	125 (86.2%)	125 (87.4%)
Soft tissue mobilisation	0.0 (0.0, 16.7)	0.0 (0.0, 23.6)
Joint movement and mobilisation—isolated	40.0 (8.1, 68.0)	47.6 (7.4, 71.3)
Facilitation muscle activity and movement	33.3 (5.9, 71.0)	34.8 (9.2, 68.2)
Positioning	6.3 (0.0, 39.4)	9.5 (0.0, 49.0)
Specific sensory input	0.0 (0.0, 13.4)	0.0 (0.0, 14.3)
Exercise to increase strength	40.9 (11.6, 74.7)	30.0 (0.0, 69.6)
Balance and mobility incorporating upper limb	59.3 (20.0, 83.3)	35.7 (0.0, 73.4)
Upper limb functional tasks	60.0 (23.9, 84.5)	40.0 (18.3, 78.9)
Education for patient or carer	10.0 (0.0, 39.4)	10.0 (0.0, 44.2)

**FST**		
Number participants with treatment records	144 (99.3%)	0
Muscle group/s specific movement	100.0 (94.4, 100.0)	0
Hand reach/retrieval activity	80.0 (56.3, 94.7)	0
Hand reach/retrieval in side lying	0.0 (0.0, 5.4)	0
Hand grip	83.3 (53.4, 96.3)	0
Hand manipulation activity	73.2 (37.6, 95.5)	0

**MPT**		
Number participants with treatment records	0	142 (99.3)
	0	
Soft tissue mobilisation	0	31.3 (0.0, 83.8)
Joint movement and mobilisation—isolated	0	100.0 (85.4, 100.0)
Facilitation of muscle activity and movement	0	75.0 (29.5, 96.4)
Positioning	0	50.9 (15.1, 91.2)
Specific sensory input	0	45.6 (10.7, 80.6)
Exercise to increase strength	0	69.2 (37.5, 91.7)
Balance and mobility incorporating upper limb	0	7.4 (0.0, 40.3)
Upper limb functional tasks	0	92.9 (77.3, 100.0)
Education for patient or carer	0	55.6 (17.9, 82.4)

### Clinical Efficacy

There were 126 participants in the FST + CPT group and 114 in the MPT + CPT group who had a total score for the primary outcome of ARAT at both baseline and outcome, i.e., all sections completed on the case report form (Table 6). By follow-up, the number of participants with full ARAT scores also at baseline had dropped to 104 for the FST + CPT group and 100 for the MPT + CPT group (Table 6). Mean (SD) ARAT change scores (baseline to outcome) were clinically important for both groups: FST + CPT = 9.70 (11.72) and MPT + CPT = 7.90 (9.18). At follow-up, further mean (SD) ARAT change scores from baseline were evident for both groups: FST + CPT = 11.10 (14.68) and MPT + CPT = 10.3 (10.74). However, at neither time point was the small between-group difference statistically significant: *p* = 0.298 at outcome and *p* = 0.743 at follow-up (Table 6).

**Table 6:** Change from baseline to outcome and follow-up for primary outcome, Action Research Arm Test score, paretic (contralesional) upper limb for participants with data at both time points.

Comparison	Treat group	Number participants^a^	Baseline: mean (SD)	Outcome: mean (SD)	Change: mean (SD)	Least squares: mean [95% confidence interval (95% CI)]	Least squares mean difference (95% CI) and *p*-value of change between group
At outcome	Functional strength training (FST) + conventional physical therapy (CPT)	126	24.40 (18.45)	34.10 (17.81)	9.70 (11.72)	9.80 (7.87, 11.73)	1.35 (−1.20, 3.90); *p* = 0.298
	Movement performance therapy (MPT) + CPT	114	26.50 (17.78)	34.40 (18.68)	7.90 (9.18)	8.45 (6.41, 10.49)	
At follow-up	FST + CPT	104	25.80 (18.21)	36.80 (19.14)	11.10 (14.68)	10.90 (8.31, 13.49)	0.55 (−2.77, 3.88); *p* = 0.743
	MPT + CPT	100	27.10 (17.49)	37.40 (17.50)	10.3 (10.74)	10.35 (7.66, 13.03)	

*^a^Number of participants with data at both time points*.

Improvements for the secondary clinical efficacy outcome measures (Table 7) showed a similar pattern to the ARAT scores (primary outcome) in that improvements happened over the timecourse of the trial for both groups. However, the small differences between the two intervention groups did not reach statistical significance.

**Table 7:** Change from baseline to outcome and follow-up for secondary outcomes, Wolf Motor Function Test performance (WMFT), grip force, and pinch force, paretic (contralesional) upper limb for participants with data at both time points.

Comparison	Treat group	Number participants^a^	Baseline: mean (SD)	Outcome: mean (SD)	Change: mean (SD)	Least squares: mean [95% confidence interval (95% CI)]	Least squares mean difference (95% CI) and *p*-value of change between group
**WMFT**							
At outcome	Functional strength training (FST) + conventional physical therapy (CPT)	117	36.60 (19.97)	47.80 (19.70)	11.20 (10.62)	11.31 (0.35, 13.27)	0.65 (−1.91, 3.21); *p* = 0.616
	Movement performance therapy (MPT) + CPT	109	39.00 (16.84)	49.00 (18.52)	10.00 (9.61)	10.65 (8.62, 12.69)	
At follow-up	FST + CPT	98	38.30 (19.42)	51.80 (19.83)	13.50 (14.28)	14.21 (11.53, 16.88)	0.01 (−3.46, 3.47); *p* = 0.997
	MPT + CPT	93	39.30 (16.96)	52.60 (18.41)	13.30 (11.55)	14.20 (11.40, 17.00)	

**Grip force**							
At outcome	FST + CPT	122	7.60 (8.72)	10.7 (9.99)	3.1 (7.11)	3.98 (2.74, 5.21)	0.47 (−1.16, 2.09); *p* = 0.571
	MPT + CPT	115	7.20 (8.19)	9.90 (9.35)	2.70 (6.25)	3.51 (2.24, 4.78)	
At follow-up	FST + CPT	101	7.40 (8.50)	11.80 (10.20)	4.4 (7.02)	5.33 (3,70, 6.95)	−0.29 (−2.37, 1.79); *p* = 0.785
	MPT + CPT	97	7.20 (8.35)	12.00 (10.10)	4.70 (8.71)	5.62 (3.95, 7.28)	

**Pinch force**							
At outcome	FST + CPT	115	2.20 (2.20)	3.00 (2.93)	0.90 (2.13)	0.91 (0.48, 1.33)	0.02 (−0.54, 0.59); *p* = 0.934
	MPT + CPT	109	2.00 (2.30)	2.90 (2.88)	0.90 (2.16)	0.89 (0.45, 1.32)	
At follow-up	FST + CPT	94	2.30 (2.22)	3.30 (2.45)	1.00 (2.19)	1.16 (0.62, 1.71)	−0.30 (−0.98, 0.39); *p* = 0.395
	MPT + CPT	95	2.10 (2.24)	3.50 (3.09)	1.40 (2.76)	1.46 (0.92, 2.01)	

*^a^Number of participants with data at both time points*.

### Neural Correlates of Clinical Improvement

Change in neural variables derived from neuroimaging between baseline and outcome for those participants with data at both visits, and ARAT data at both visits are shown in Table 8. There were no statistically significant differences between the two groups for change in any of these variables. Change between baseline and outcome for RMT for pBB and pECR muscles for those participants with data at both visits and ARAT data at both visits is shown in Table 8. There were no statistically significant differences between the two groups for change in either of these variables.

**Table 8:** Change (outcome—baseline) in neural variables for participants with data at both visits for functional strength training (FST) + conventional physical therapy (CPT) and movement performance therapy (MPT) + CPT groups.

Variable	Group	*N*	Baseline: mean (SD)	Outcome: mean (SD)	Change: mean (SD)	Least squares: mean (95% CI)	Least squares mean difference (95% CI), *p*-value
Lesion volume, mm^3^ (log-transformed)	FST + CPT	24	8.20 (2.13)	8.10 (2.17)	−0.10 (0.45)	−0.11 (−0.31, 0.09)	−0.12 (−0.36, 0.13); *p* = 0.349
	MPT + CPT	20	8.20 (2.49)	8.10 (2.40)	−0.00 (0.32)	0.01 (−0.21, 0.22)	
Corticocortical connectivity^a^	FST + CPT	20	0.38 (0.09)	0.38 (0.09)	−0.00 (0.02)	−0.01 (−0.02, 0.01)	0.01 (−0.01, 0.03); *p* = 0.386
	MPT + CPT	18	0.45 (0.07)	0.43 (0.08)	−0.01 (0.03)	−0.02 (−0.03, −0.00)	
Corticospinal connectivity^b^	FST + CPT	20	0.03 (0.05)	0.03 (0.04)	0.00 (0.01)	0.00 (−0.01, 0.02)	−0.01 (−0.02, 0.01); *p* = 0.524
	MPT + CPT	18	0.01 (0.05)	0.031 (0.05)	0.02 (0.04)	0.01 (−0.01, 0.03)	
RMT pBB	FST + CPT	28	61.6 (12.07)	60.6 (13.61)	−1.0 (14.27)	−0.82 (−6.32, 4.68)	−2.87 (−8.91, 3.16); *p* = 0.343
	MPT + CPT	27	62.5 (13.83)	65.0 (14.90)	2.5 (10.06)	2.06 (−3.86, 7.97)	
RMT pECR	FST + CPT	28	53.1 (13.15)	52.0 (11.62)	−1.1 (11.46)	−3.29 (−7.68, 1.09)	0.98 (−4.31, 6.28); *p* = 0.710
	MPT + CPT	27	57.4 (17.16)	53.2 (12.93)	−4.2 (12.98)	−4.28 (−9.19, 0.63)	

*RMT pBB, resting motor threshold paretic Biceps brachii; RMT pECR, resting motor threshold paretic extensor carpi radialis; 95% CI, 95% confidence interval*.

*^a^Fractional anisotropy MNI corpus callosum*.

*^b^Asymmetry ipsilesional:contralesional Montreal Neurological Institute corticospinal tract*.

The correlations between baseline to outcome change scores for neural variables and ARAT scores ranged from *r* = −0.001 (*p* = 0.996) for RMT pECR (all participants) to *r* = −0.306 (*p* = 0.232) for the MPT + CPT group for corticospinal anatomical connectivity (Table 9). No significant correlations between ARAT improvement and neural measures were identified in either group or overall.

**Table 9:** Correlations between change (from baseline to outcome) in neural correlates and paretic ARAT total score (primary outcome measure).

	FST + CPT			MPT + CPT			All		
	Number	*r*-Value	*p*-Value	Number	*r*-Value	*p*-Value	Number	*r*-Value	*p*-Value
Volume of stroke lesion	24	−0.021	0.921	19	−0.043	0.863	43	−0.042	0.787
Corticocortical anatomical connectivity	20	0.092	0.699	17	−0.029	0.913	37	0.069	0.684
Corticospinal anatomical connectivity	20	−0.031	0.897	17	−0.306	0.232	37	−0.147	0.385
RMT—pBB	28	0.095	0.631	27	0.199	0.320	55	0.093	0.501
RMT—pECR	28	−0.080	0.635	27	0.043	0.831	55	−0.001	0.996

*CST, corticospinal tract; pBB, paretic biceps brachii; pECR, paretic extensor carpi radialis muscle; FST, functional strength training; CPT, conventional physical therapy; MPT, movement performance therapy; ARAT, Action Research Arm Test; RMT, resting motor threshold*.

### Predictive Neural Markers of Clinical Improvement

For those people with both ARAT and neural data at baseline and outcome, there were no statistically significant interaction effects (Table 10).

**Table 10:** Subgroup analysis of interaction effect between baseline neural variables and change in paretic Action Research Arm Test (ARAT) total score from baseline to outcome.

		FST + CPT paretic ARAT			MPT + CPT paretic ARAT			Least squares mean difference (95% CI)	Interaction *p*-value
		*N*	Baseline: mean (SD)	Change at outcome: mean (SD)	*N*	Baseline: mean (D)	Change at outcome: mean (SD)		
MNI CST affected^a^	No	5	27.0 (17.07)	17.2 (13.65)	7	27.6 (23.59)	6.1 (7.13)	20.64 (−14.07, 55.36)	0.384
	Yes	33	23.3 (19.67)	11.9 (12.32)	27	25.7 (17.04)	8.3 (7.40)	2.88 (−2.29, 8.04)	

Volume of stroke lesion (logged)	<Median	17	25.1 (18.64)	16.1 (11.99)	20	28.7 (18.96)	9.7 (8.03)	5.30 (−1.09, 11.70)	0.762
	≥Median	21	22.8 (19.99)	9.8 (12.36)	14	22.4 (16.98)	5.4 (5.39)	4.44 (−2.89, 11.76)	

FA MNI corpus callosum midline^b^	<Median	18	27.4 (20.29)	8.2 (9.47)	14	27.4 (17.53)	6.6 (6.87)	3.83 (−2.41, 10.07)	0.723
	≥Median	18	22.3 (18.22)	17.1 (13.45)	18	24.6 (17.74)	9.2 (6.78)	4.29 (−2.99, 11.58)	

Asymmetry MNI CST^c^^,^^d^	<Median	19	28.6 (16.69)	14.6 (11.90)	16	31.9 (15.80)	10.4 (6.65)	2.42 (−3.95, 8.79)	0.553
	≥Median	17	20.6 (21.36)	10.4 (12.73)	16	19.7 (17.26)	5.7 (6.35)	6.62 (−0.33, 13.56)	

Presence of MEP pBB^e^	No	16	12.6 (15.34)	9.4 (11.43)	13	12.7 (13.31)	9.5 (7.41)	−0.60 (−7.38, 6.18)	0.237
	Yes	34	32.2 (16.69)	10.9 (11.85)	36	34.0 (15.05)	6.2 (7.95)	3.19 (−0.71, 7.09)	

pBB resting motor threshold	<Median	15	37.8 (12.86)	9.3 (8.83)	16	36.6 (15.29)	5.7 (7.37)	3.17 (−2.27, 8.62)	0.697
	≥Median	19	27.7 (18.29)	12.1 (13.90)	20	32.0 (14.92)	6.7 (8.54)	2.89 (−3.11, 8.88)	

Presence of MEP pECR^f^	No	14	10.4 (13.15)	7.1 (8.98)	11	9.7 (12.62)	8.1 (9.31)	−1.74 (−9.54, 6.07)	0.193
	Yes	36	31.9 (16.87)	11.7 (12.37)	38	33.8 (14.60)	6.8 (7.52)	3.41 (−0.53, 7.34)	

pECR resting motor threshold	<Median	18	35.6 (16.02)	12.1 (11.41)	15	40.2 (8.39)	5.9 (6.19)	2.30 (−1.74, 6.34)	0.503
	≥Median	18	28.3 (17.35)	11.3 (13.58)	22	29.5 (16.71)	7.6 (8.47)	2.57 (−4.06, 9.21)	

*^a^Corticocortical anatomical connectivity*.

*^b^FA = Fractional anisotropy*.

*^c^Corticocortical anatomical connectivity (ipsilesional:contralesional)*.

*^d^CST = Corticospinal tract*.

*^e^MEP pBB = Motor evoked potential paretic biceps brachii*.

*^f^pECR = Paretic extensor carpi radialis*.

### Adverse Reactions and Adverse Events

Four participants in the FST + CPT group experienced an adverse reaction (for pain *n* = 1, and for fatigue *n* = 3). Two people in the MPT + CPT group experienced an adverse reaction (for pain *n* = 1, and for fatigue *n* = 1).

Adverse events were experienced by 61 (42.1%) of participants in the FST + CPT group and 68 (47.6%) of participants in the MPT + CPT group. Serious adverse events were experienced by 26 (18.0%) of participants in the FST + CPT group and 15 (10.5%) in the MPT + CPT group. Serious adverse events in the MedDRA System Organ Class “nervous system disorders” were experienced by six (4.1%) of participants in the FST + CPT group and two (1.4%) of people in the MPT + CPT group.

### Health Economics

#### Intervention Cost

The FST equipment cost was estimated to be £898.74; when divided across the 145 participants allocated to the FST intervention this equates to £6.20 per participant (Table 11). A total of eight FST training workshops took place, where the length varied between 1 and 10 h (the latter was spread over 2 days). Trainer time was estimated as a cost of £3,782 (61 h in total, including 8 h preparation); trainee time was estimated as £4,488 (102 h in total; and travel, accommodation and room hire costs were estimated at £7,016). This equated to a total of £15,286, or an estimated £105.42 per FST participant (Table 11).

**Table 11:** Levels of resource use (available case, per participant over the 6-month follow-up period) and mean participant costs (£) (complete case participants unadjusted).

	Resource use		Participant costs		
	FST + CPT (*N* = 145)	MPT + CPT (*N* = 143)	FST + CPT (*N* = 56)	MPT + CPT (*N* = 143)	*p*-Value
Visits from health professionals visit (mean)	108.84 (500.78) (*n* = 102)	57.81 (63.56) (*n* = 100)	2,725.81 (2,315.29)	2,719.03 (2,381.39)	0.900
Occupational therapist	9.87 (17.25) (*n* = 102)	8.52 (11.58) (*n* = 100)	553.84 (795.33)	548.76 (703.74)	0.971
Social worker	0.73 (1.37) (*n* = 102)	0.86 (1.50) (*n* = 100)	54.89 (107.72)	103.73 (186.70)	0.083
Speech and language therapist	2.45 (6.78) (*n* = 102)	1.97 (5.75) (*n* = 100)	186.68 (525.73)	180.36 (522.09)	0.948
Nurse	3.25 (6.63) (*n* = 102)	5.01 (12.54) (*n* = 100)	82.56 (186.25)	115.71 (251.76)	0.423
GP	2.73 (4.95) (*n* = 102)	2.10 (1.85) (*n* = 100)	106.77 (86.24)	104.26 (90.85)	0.879
Community care assistant	69.17^a^ (502.73) (*n* = 102)	17.50 (54.71) (*n* = 100)	448.67 (1,404.37)	191.98 (471.15)	0.197
Physiotherapist	18.19 (21.08) (*n* = 102)	19.78 (19.92) (*n* = 100)	1,265.27 (1,201.70)	1,314.77 (1,409.25)	0.839
Other	2.47 (13.68) (*n* = 102)	2.07 (12.48) (*n* = 100)	75.13 (444.44)	159.45 (1,123.31)	0.600
Hospital admissions (mean bed days)	9.15 (21.82) (*n* = 92)	10.49 (25.36) (*n* = 91)	2,625.74 (6,018.34)	4,259.14 (8,606.40)	0.234
Accident and emergency (mean visits)	0.25 (0.70) (*n* = 102)	0.32 (0.66) (*n* = 103)	13.76 (34.02)	26.85 (61.27)	0.161
Alternative residences (mean weeks)	0.57 (2.36) (*n* = 102)	0.45 (2.62) (*n* = 103)	4.35 (32.55)	364.32 (2,447.58)	0.274
Nursing home	0.00 (0.00) (*n* = 102)	0.06 (0.59) (*n* = 103)			
Residential care	0.51 (2.30) (*n* = 102)	0.35 (2.53) (*n* = 103)^b^			
Other	0.06 (0.59) (*n* = 102)	0.05 (0.41) (*n* = 103)			
NHS/PSS professional carer (mean hours)	2.68 (3.90) (*n* = 86)	1.74 (4.82) (*n* = 91)	2,602.14 (12,774.31)	1,022.75 (2,964.16) (134.30)	0.350
Prescriptions (mean number)	4.82 (3.01) (*n* = 78)	4.81 (3.00) (*n* = 78)	315.61 (1,162.04)	175.03 (242.30)	0.358
FST training			105.42 (0.00)	0	<0.001
FST equipment			6.20 (0.00)	0	<0.001
Overall NHS and PSS costs			8,447.03 (13,816.73)	8,567.13 (9,623.58)	0.977

*N, Number of patients in receipt; n, number of patient for whom data were available; PSS, Personal Social Services; FST, functional strength training; CPT, conventional physical therapy; MPT, movement performance therapy*.

*^a^This includes 5,040 visits that were reported by 1 participant. If these data were excluded the mean value would be 19.95*.

*^b^One participant reported that they spent 5 months in residential care*.

#### Other NHS and PSS Costs

Details of the available resource use data are provided in Table 11. The unit costs that were assigned to the resource use items, to produce the cost estimates are reported in Table 12. Details of medication (prescribed by the doctor) were provided by 78 (54.5%) of the 143 MPT + CPT participants, and by 78 (53.8%) of the 145 FST + CPT participants. Participants in both groups reported a considerable number of visits from health professional—a mean of 58 per participant over the 6-month follow-up period in the MPT + CPT group, compared to 109 per participant for the FST + CPT group. Indeed, visits from health professional, and hospital admission costs, were found to be the two highest cost components, whereas, for example, the total A&E visit cost was much lower. However, it should be noted that these values can be skewed by a few reported high values (Table 11).

**Table 12:** Quality of life outcomes (available case, per participant).

item mean score (SD) (*n*)	FST + CPT (*N* = 145)	MPT + CPT (*N* = 143)	*p*-Value
Baseline EQ-5D-3L score	0.428 (0.332) (*n* = 140)	0.421 (0.326) (*n* = 143)	0.860
6-week EQ-5D-3L score	0.523 (0.308) (*n* = 124)	0.508 (0.324) (*n* = 117)	0.728
6-month EQ-5D-3L score	0.566 (0.297) (*n* = 103)	0.562 (0.313) (*n* = 99)	0.939
EQ-5D-3L 6-month change score	0.131 (0.305) (*n* = 101)	0.121 (0.301) (*n* = 99)	0.815
QALY score (over 6 months)	0.266 (0.134) (*n* = 101)	0.266 (0.137) (*n* = 99)	0.995

*n, Number for whom data were available; QALY, Quality-Adjusted Life Years; FST, functional strength training; CPT, conventional physical therapy; MPT, movement performance therapy*.

#### Quality of Life Outcomes

The EQ-5D-3L scores were slightly higher for the FST + CPT group at baseline, 6-week and 6-month follow-up (Table 12). Over the 6 months both the MPT + CPT and FST + CPT group showed a mean increase of 0.121 and 0.131, respectively (Table 12). There was no significant difference between the groups.

#### Cost-Effectiveness

The results of the base-case bivariate regression are shown in Table 13, where it can be seen that FST + CPT was estimated to be associated with a lower cost and slightly lower QALY score. The incremental cost (95% CIs) was −£180.35 (−£48,388.28, £4,027.58). The associated net-benefit was £114 at an (λ) of £20,000 per QALY. However, neither the difference in mean cost nor the difference in mean effect was statistically significant. Similarly the CEAC suggests there is a >40% chance of making the wrong decision by choosing FST + CPT at an (λ) of £20,000 per QALY. This demonstrates there is considerable uncertainty associated with this result.

**Table 13:** Base-case and sensitivity analyses: incremental cost, incremental effect, and cost-effectiveness of FST + CPT versus MPT + CPT.

Analysis (Nm, Nf)	Incremental cost (95% CI) (£)	Incremental effect (95% CI)	ICER/net benefit^a^ (£)	CEAC± (%)

	QALY gain			
Base-case: complete case (61, 56)	−236.89 (−4,444.81 to 3,971.03)	−0.0018 (−0.037 to 0.0330)	200.59^a^	58.9
SA1: imputed (143, 145)	1,205.60 (−2,196.60 to 4,613.80)	0.0055 (−0.0232 to 0.034)	219,157.06	27.2
SA2: excluding intervention training/equipment costs (61, 56)	−348.51 (−4,556.43 to 3,859.42)	−0.002 (−0.037 to 0.033)	312.21^a^	60.1
SA3: including carer costs (paid for privately) (61, 56)	−784.75 (−5,712.80 to 4,143.29)	−0.002 (−0.037 to 0.033)	747.94^a^	63.9
SA4: including carer costs (paid for privately) plus any other help/care (61, 56)	3,757.301 (−7,598.65 to 15,113.20)	−0.002 (−0.037 to 0.033)	−3,795.91^a^	30.7
SA4b: including carer costs (paid for privately) plus any other help/care, with inconceivable outliers excluded^b^ (60, 55)	−392.10 (−6,081.78 to 5,297.58)	−0.004 (−0.039 to 0.031)	310.43^a^	54.5
SA5: winsoring (53, 54)	−506.80 (−3,038.42 to 2,024.81)	−0.001 (−0.038 to 0.036)	488.01^a^	64.0

*95% CI, 95% confidence interval; ICER, incremental cost-effectiveness ratio; Dominant, lower mean costs and higher mean effect; QALY, Quality-Adjusted Life Years; FST, functional strength training; CPT, conventional physical therapy; MPT, movement performance therapy*.

*Nm and Nf equal the number included in the analysis from the MPT + CPT and FST + CPT groups*.

*SA1, …, SA5 refer to the different sensitivity analyses described in Section Materials and Methods; QALY over 6 months; ±probability of bring cost-effective on the cost-effectiveness acceptability curve (CEAC) at the threshold (λ) of £20,000 per QALY*.

*^a^Denotes only net benefit was reported, ICER not reported as either a negative cost or effect was estimated*.

*^b^Two participants stated a length of care time which exceeded the timeframe in question, i.e., they reported more hours than there are in a week and were dropped from SA4b*.

#### Sensitivity Analyses

The results of the five sensitivity analyses are shown in Table 13. In line with the base-case results, there were no significant differences between the mean costs or mean effects between groups. Similarly, the probability of making the wrong decision by choosing a particular option was always >25% according to the CEAC [at an (λ) of £20,000 per QALY], again suggesting that there is considerable uncertainty.

## Discussion

### Summary of Main Results

#### Clinical Efficacy

Small differences between the groups in upper limb recovery were found for some measures, but, these were neither clinically important nor statistically significant. The variability within groups was considerable especially for (hand) grip force and (finger) pinch force.

#### Neural Correlates of Clinical Improvement

This study found no clinically important association between clinical improvement and change in the neural measures in response to either trial intervention. However, the sample sizes available for analysis of neural–therapy interactions were small for reasons including: lack of consent for these measures; lack of equipment availability; contraindications to MRI and/or TMS; and a participant being unable to attend a measurement session. In addition, the simple neural measures that are the focus of this report may not capture the most relevant or sensitive features of residual brain structure and function.

#### Predictive Markers of Clinical Improvement

This study found no interaction effects between baseline neural variables and change in ARAT total score (primary outcome measure) for the more paretic (contralesional) upper limb over a 6-week intervention (between baseline and the primary outcome time point) for people recruited from consecutive admissions to stroke services. In common with the neural correlates of clinical improvement analysis the sample sizes available for analysis of neural–therapy interactions were smaller than anticipated.

#### Estimate of Cost-Effectiveness

There were no significant differences in mean cost or mean QALY score between the groups.

### Strengths and Limitations

Bias protection for this trial was provided by concealment of randomisation order and group allocation *via* an independent telephone randomisation service, and reporting all planned outcomes. Importantly, trial procedures included blinding of assessors to group allocation. However, because of staffing challenges in centres at some points in the trial it was not always possible to have blinded assessment of the behavioural outcomes (ARAT, WMFT, grip force, and pinch force). Moreover, because both experimental interventions were behavioural, it was not possible to blind research therapists or participants to group allocation. Although non-blinding to group allocation is a limitation for trials of pharmaceutical agents, blinding is not always possible in trials of many stroke rehabilitation interventions [e.g., Ref. (61, 91)]. However, to maximise the blinding of clinical staff to participants’ group allocation, the research team did not reveal which intervention was allocated and which research therapist was delivering which experimental intervention.

The sample size for this trial was estimated by a power calculation informed by data from an early phase trial of FST + CPT and MPT (CPT) + CPT (32). The estimated sample size allowed for an attrition rate of 10% at the primary time point of outcome. In the event, our attrition rate at outcome was 12.5% which is substantially lower than the 25% reported by the EXCITE Trial (92), similar to the 12.7% reported by the ICARE trial (61) and just below the 14% reported by the EVREST trial (91). A strength of the power calculation was that it considered the anticipated clustering of participants within therapists. However, staff turnover resulted in more therapists providing either MPT or FST for participants than was envisaged originally. Although this limited the level of clustering of participants within therapists, it is also a strength because a larger number of therapists provides greater generalisability to clinical practice.

At baseline, 59% of participants were recruited within 30 days of stroke and 41% recruited at 31 days or more (Table 2). To minimse potential differences in response to therapy the randomisation process considered this in the stratification. This was successful at balancing time after stroke between the groups (Table 2).

The recruitment rate for this FAST-INdiCATE trial was 5.7% of those screened, which is within the range of those reported for recent respected trials of physical therapy interventions in the first 3 months after stroke, e.g., 3.3% (61), 8.3% (93), and 15.8% (91).

This trial was congruent with the requirements for stroke rehabilitation trials (21, 62, 94). A key strength for all aspects of this trial is that the experimental interventions, both MPT and FST, were described in sufficient detail to enable both research replication of the trial and transferability of results to clinical practice (15, 95, 96). Thus, this trial has followed the recommendations for improving rehabilitation research (15, 94, 97). A particular strength of this trial is the complete reporting of the content and dose of therapy that was actually provided, including the CPT, since the majority of stroke rehabilitation studies do not report fidelity to intended therapy (91, 97). When information is reported it is usually in terms of dose rather than content (61).

Another important strength of this trial is that 59% of participants provided informed consent within 30 days of stroke. Early rehabilitation is recommended because the brain has most potential for reorganisation, particularly in the first month after stroke, but also in the following 2 months (9). In clinical practice, most rehabilitation is provided in this early period, and yet most rehabilitation trials are conducted later in recovery (21, 98). Moreover, participants in this trial were representative of those who receive upper limb rehabilitation in clinical stroke services and not just those with moderate or mild paresis. The results are therefore directly relevant to clinical practice.

A key strength of this trial is that mechanistic studies were integral to the design. In addition, the present trial has addressed the need to investigate both the underlying mechanisms of effect of stroke rehabilitation interventions and which people are most likely to respond to which interventions (16, 17, 21, 99). Thus, unlike many mechanistic studies, those underpinning this study guarded against potential risk of bias and allowed for sufficient description of the interventions provided.

However, disappointingly, analysis of the neural correlates study was restricted by the unanticipated low proportion of participants who had both (a) full sets of clinical data and (b) neural measures at the key time points of baseline and outcome. This ranged from 37 participants for corticocortical anatomical connectivity to 55 participants for RMT in pBB and pECR muscles. Likewise, the predictive markers analysis was also restricted by the smaller than expected number of participants. Nevertheless, although the number of participants was lower than anticipated initially, there were still substantially more than in many other studies. Other studies have also reported challenges in undertaking TMS measurements in stroke survivors, with TMS measures reported to have been collected in 33% of a sample of participants recruited from a database 4–20 months after stroke (100), which is identical to that in this trial (Table 1). Unlike the well-regarded PREP algorithm development study (27), participants with contraindications to MRI and TMS were not excluded from this study. Consequently the smaller than expected number of participants with neural measures is likely to be a consequence of embedding mechanistic studies into a pragmatic clinical trial. In addition, the multicentre design of this study means that different scanners were used at different sites and some variation in scanner protocols was unavoidable. This has the potential to introduce between-site variability into the imaging-derived metrics that is not taken into account in our analysis. Yet, this trial demonstrates that mechanistic neural studies can be embedded in pragmatic clinical trials. Furthermore, this trial shows that highly specialised neurophysical and imaging-based measures can be made outside of specialist neurological research centres. The sites for this trial had not conducted these mechanistic investigations before. However, neural data collection was not problem free, as evidenced by the number of measures not undertaken for reasons such as (Table 1): not giving informed consent for MRI (19.4% at baseline and 26.4% at outcome); not giving informed consent for TMS (12.8% at baseline and 15.6% at outcome); unavailability of the MR scanner (5.9% at baseline and 6.0% at outcome); and unavailability of TMS (8.3% at baseline and 5.8% at outcome).

The inclusion of one of the few health economic investigations of well-defined stroke rehabilitation interventions further adds strength to this trial. We have identified large cost drivers (i.e., visits from health professionals and hospital admissions) and also resources that have a relatively low cost, e.g. A&E, visits. There is therefore an argument for not including such resource items in future studies since they are unlikely to differ between groups and, in turn, this would hopefully improve data completeness.

### Relationship to Previous Studies

#### Therapy Received by Participants

It is reassuring that the dose and content of CPT were essentially the same for the two experimental groups but these data indicated considerable variance around the mean values for most categories of therapy, irrespective of type (Table 5). It is possible that this variance is due to differences between participants, and that this influenced clinical therapists’ choice of therapy category. It is also possible that variance is due to differences between therapists. Further investigation of these data will be undertaken and reported in a future publication.

Examination of the content of the two experimental interventions could lead to the interpretation that there was overlap, since 69% of the sessions for the MPT + CPT group included “exercise to increase strength,” and 93% included upper limb functional tasks. However, the distinction between the content of FST and MPT was maintained by adherence to protocol, namely, that: (a) any functional tasks and resistance training provided within MPT was neither repetitive nor progressed systematically and (b) the focus was on intrinsic feedback on movement performance through verbal and hands-on techniques. Monitoring of adherence to protocol throughout the trial was through training sessions and inter-team consultations. In this way, any uncertainties about what constituted both MPT and FST were addressed with direct reference to the therapy protocols. Although it would have been useful to collect more detailed information about the precise techniques provided and time spent on each within the therapy categories, this was not possible for pragmatic reasons. However, the description of physical therapy interventions provided in this present report is much more detailed and replicable than the “black box” descriptions of only a few years ago (101, 102).

The protocol stated that participants would be provided with up to 90 min of their allocated experimental therapy, up to 5 days a week for up to 6 weeks. This was a potential dose of 45 h in 30 sessions. In the event, mean (SD) hours of therapy were 14.8 (9.9) and 19.2 (11.1) for all participants randomised to the FST + CPT and MPT + CPT groups, respectively. Most participants were not able to tolerate 90 min in one session, fatigue being the most frequent specific single reason (Table 4). The therapy dose reported here is lower than the dose of therapy delivered in a preliminary dose-ranging trial of arm training (103). However, the dose ranging trial recruited 32 people who were: “able to tolerate the interventions and evaluations”; following their first-ever stroke; and were aged a mean (SD) of 50 (12) years. Whereas the data reported here were for all randomised participants, aged approximately 20 years older than in the dose-ranging trial, from a consecutive series of admissions to stroke services, and not just those who were judged to be able to tolerate a high dose of therapy. So, the present trial is more likely to be representative of current routine clinical practice. Furthermore, data in this report relate not just to those participants who remained in the trial at outcome and/or follow-up (Table 4). Planned subsequent analyses will explore whether higher doses were delivered to those still in the trial at the outcome time point. Although most participants were not able to tolerate 90 min in one session they were able to participate in sessions lasting a mean (SD) of 46.2 (16.9) and 57.6 (17.9) min for the FST + CPT and MPT + CPT groups, respectively. This is similar to the 60-min sessions delivered to approximately 77% of participants in the ICARE trial (61). When the dose of CPT is added to experimental therapy then participants in this trial received a similar dose of physical therapy to that received by ICARE trial participants (61).

#### Clinical Efficacy

The results of this trial differ from the early phase findings that suggested a trend towards better upper limb recovery, as assessed by ARAT score, in response to FST + CPT compared to MPT + CPT early after stroke (32). However, the findings of the present trial, with a larger sample size, showed no significant difference between FST + CPT and MPT + CPT in respect of improvement of ARAT score over the 6-week intervention phase. It is possible that a difference could have been detected if motor impairment had also been measured; however, in the interests of minimising burden to participants, we did not undertake these measures.

Dose of physical therapy could have been a confounding factor for the present results as it has been associated with enhanced outcome [e.g., Ref. (21, 58)]. However, there are systematic review findings suggesting that that different forms of physical therapy provided to participants in the higher and lower dose groups may confound any association (67). Consequently, the association (Pearson’s correlation coefficient) between the total dose of CPT plus either FST or MPT was scrutinised to check for influence on response to therapy dose received. Since no dose effect on response to either form of therapy was discernible (Figure 2), it appears unlikely that dose of therapy confounded the finding of no statistically significant difference between FST + CPT and MPT + CPT. Furthermore, there was no between-group difference in either the dose or the content of CPT.

That MPT + CPT was found to produce equivalent benefit to FST + CPT is interesting, since FST is based on findings that task-specific training drives recovery after stroke (104), and that the largest impact on upper limb improvement is loss of muscle strength (19, 20). By contrast, MPT concentrates on enhancing the quality of movement during whole or part functional tasks and not on repetitive progressive training of those everyday tasks. Equivalence of effect is not expected from interpretations of evidence indicating that therapy should be repetitive and task-orientated/specific (105, 106). However, a recent systematic review concludes that the evidence for repetitive task training is of low quality (7), the ICARE trial of task-orientated upper limb rehabilitation found that recovery was equivalent to an equal dose of therapy-as-usual for people recruited a mean of 46 days after stroke (61), and an overview of systematic reviews included interventions for sensory impairment in the list of interventions for which there was moderate-quality evidence of benefit (107). In addition, there is some evidence of effect of such impairment-based therapy (43) and a meta-analysis found that conceptually different physical therapies have equal efficacy (4). It appears therefore that MPT is equally as beneficial as FST when given in addition to CPT.

Noticeable from these results is that there is substantial variation around the mean change from baseline for both FST + CPT and MPT + CPT (Table 6). So, the present findings support the concept underlying the scientific driver for this trial, namely: that the population of stroke survivors exhibit interindividual differences in recovery (8, 27) and may respond differently to different physical therapies (15, 20). This was not unexpected as it has been reported for other trials of physical therapies [e.g., Ref. (91)] and recognised as an important area for rehabilitation research (21, 25).

#### Predictive Markers of Response

The trial reported here is most likely one of the first trials of people recruited from consecutive admissions to a stroke service and designed to identify predictive markers of response to specific physical therapies early after stroke. Indeed, only six randomised controlled trials were included in a recent systematic review of predictive markers, none of which investigated stroke rehabilitation (108).

It was anticipated that data from the embedded investigation of predictive markers of response would provide some explanation of the variation of response to the experimental therapies (25, 109). Indeed, previous studies have indicated that combining neuroimaging and neurophysiological measures such as corticospinal tract integrity (DTI), corpus callosum connectivity (DTI), presence of a motor-evoked potential in response to TMS, corticocortical connectivity, and corticospinal connectivity may be predictive markers of recovery (25, 110). These observations have been used in the PREP algorithm to enable prediction of recovery at 12 weeks after stroke (27). Comparison of the present trial with the PREP algorithm indicates several reasons why findings might differ.

First, the time points for outcome were different. The PREP algorithm begins assessment at 72 h after stroke with clinical measures of ability to contract contralesional muscle. Then at 2 weeks after stroke, those stroke survivors not scoring at least 80% on the clinical measure undertake TMS measures for presence of an MEP in ECR and if this is absent then MRI is undertaken for DTI measurement of the structural integrity of the corticospinal pathway. In the trial reported here, most measures were undertaken later, with 41% providing informed consent at 31 or more days after stroke. It is possible therefore that measurement undertaken more than 2 weeks after stroke has less predictive power.

Second, the interventions could have been different. Although it is clear that the experimental interventions in both investigations were delivered in addition to routine therapy information about the content and dose of experimental therapy delivered to participants in the PREP study is not provided.

Third, there is a difference between definitions of outcome. The present trial was concerned with predicting response to therapy, change in ARAT score over a 6-week intervention phase; whereas the PREP algorithm has been developed to predict actual ARAT score at 12 weeks after stroke. Further investigation of data from the present trial will not be able to investigate this possibility because of the different measurement time points to those used in the PREP algorithm development study.

There have been some other investigations of predictive markers but their findings are of limited benefit for use with people early after stroke because of: lack of a comparator group; lack of specification of the therapy; participants with higher ability for voluntary movement than those in this trial, participants younger than those in this trial, and/or participants in the chronic phase after stroke (26, 109, 111, 112). Secondary analysis of the movement, MR and TMS measures undertaken in the present trial may therefore provide insight into predictive markers of response. This secondary investigation will be reported in subsequent publications.

#### Neural Correlates of Improvement

Although there have been reports of differences in neural changes in response to physical therapies which vary in efficacy (113–116), the current trial found no difference in the neural correlates of response between the experimental therapies of FST and MPT (Tables 8 and 9). This could be because no statistically significant differences were found between the two groups for any of the clinical efficacy measures (Tables 6 and 7).

Another explanation for the finding of no difference in the neural correlates of response to FST and MPT in the present trial is that previous studies were undertaken with smaller sample sizes, e.g., *n* = 13 (113), *n* = 12 (114), *n* = 14 (115), and *n* = 23 (116), thus increasing the chances of false positive findings. These compare with the sample sizes in the present trial for the MRI variables, *n* = 38, and the TMS variables, *n* = 55. In addition, the earlier studies have potential risk of bias respecting: randomisation process (113–115); observer-blind assessments (113, 114); selective recruitment (114, 115); and from results emanating from a *post hoc* analysis of an RCT (116). However, there may be genuine associations which are too subtle to identify with the available sample sizes in this trial.

It is possible that the neurophysiological mechanisms underlying therapy-induced improvements are more discrete than can be identified by the simple TMS or MR-based measures reported here. For example, an early phase investigation of potential underlying mechanisms of therapy-induced improvements found variation between individuals, which could be influenced by the level of residual voluntary motor activity capacity (117). People with lower motor activity capacity during the early period of therapy were more likely to have fewer muscle synergies and more co-contraction than people with higher motor activity capacity (117). By the end of the therapy period, those participants with lower motor activity capacity initially had increased the number of muscle synergies underlying functional movement (117). The present trial did not measure muscle synergies.

An alternative reason for lack of correlation between change in the neurophysiology measures and change in upper limb activity capacity could be because of interindividual differences in response to various forms on non-invasive brain stimulation, including TMS, in both people after stroke and neurologically intact adults (118–120). Furthermore, comparison with other studies is limited as amongst TMS studies there has been variation in the protocols used to derive the neural correlates with differences in the method of collecting MEPs (recruitment curve or Block of MEPs) and characteristics of recruitment curve (intensities of stimulation, number of stimuli, increments of intensity, and number of intensities). All these protocol differences may lead to lack of comparability across studies.

Turning to the MR data, very simple measures, which are realistic to quantity in a clinical setting, have been the focus of the current report. However, the MR data acquired here could also be interrogated with more sophisticated analysis approaches. Future reports will determine whether such approaches offer increased sensitivity to clinically relevant neural change or prediction of response to therapy.

#### Estimated Cost-Effectiveness

We are only aware of one previous study that has investigated the cost-effectiveness of FST poststroke (121). Chan (121) developed a decision analytical model to estimate the costs and benefits associated with CPT + FST over a 2-year period, where the comparators were both another increased therapy intervention (CPT + CPT) and CPT alone. Overall, in the earlier study CPT + FST was estimated to be more cost-effective than CPT + CPT, largely because the improvement in EQ-5D scores was estimated to be higher for those in receipt of FST (41). Chan (121) based the EQ-5D scores on the work by Cooke and colleagues (41), and our present results are in line with these in that we found both groups had an improvement in EQ-5D scores. However, the earlier investigation (41) found that the size of improvement (based on median scores and a smaller sample than in our study) was greater for CPT + FST compared to CPT + CPT, although in keeping with our results there was no significant difference between groups. It is difficult to compare our cost estimates to those of Chan (121), as (for the reasons outlined earlier) we have not explicitly estimated therapy costs. However, in keeping with our results, Chan (121) concluded that there was a large level of uncertainty associated with results.

### Generalisability

The generalisability of this trial is considered in the wide inclusion criteria with direct relevance to clinical practice and the conduction of the trial in three different clinical centres.

An economic evaluation, where the costs and benefits of CPT + MPT were compared to those for CPT + FST, was undertaken. In addition, the estimation of cost-effectiveness is an iterative process, and early information on costs and effects can be used to inform the design of subsequent phase III studies.

## Conclusion

The trial reported here combined investigation of the underlying mechanisms of motor recovery and predictive markers of beneficial response in a representative sample of people early after stroke with substantial to moderate upper limb motor impairment receiving well-characterised physical therapies. There were small differences in the clinical efficacy of upper limb recovery between FST and MPT given in addition to CPT but these did not reach statistical significance. Both groups showed increases in ARAT score (primary outcome measure) above the clinically important change. However, variation around the mean change from baseline scores was substantial in both groups.

The finding of no difference between two such conceptually different physical therapies is not congruent with clinical guidelines (106) and neuroscience principles (9) both of which point to advantages of task-specific training such as FST. The findings presented here, together with the results of the ICARE Trial (61), question the current emphasis on high-dose task-specific training early after stroke, particularly since there were no significant differences in mean cost or mean QALY score between the two groups.

The substantial variation around the mean improvement from baseline scores was expected from the previous early phase trials (32, 41), hence designing the trial reported here to embed explanatory neural investigation of FST and MPT for (a) predictive markers of response and (b) neural correlates of response. The findings were that none of the pretreatment neural characteristics of interest predicted response to either FST or MPT; and that the neural correlates of change were similar for the two forms of physical therapy. Consequently, there is still an urgent need for evidence to guide clinical decisions about: (a) appropriate prescription of physical therapy for individuals and (b) the recovery mechanisms at which physical therapy should be targeted (15, 16, 99).

## Ethics Statement

The protocol was approved by the Norfolk Research Ethics Service (reference number is 11/EE/0524). All participants provided informed consent.

## Author Note

The EME Programme is funded by the MRC and NIHR, with contributions from the CSO in Scotland and NISCHR in Wales and the HSC R&D Division, Public Health Agency in Northern Ireland.

## Author Contributions

SH: contributed to design of the trial and acquisition and interpretation of data; led the development of the protocol for one form of experimental intervention, training workshops for research therapists and blinded assessors, and neurophysiology data collection at one clinical centre; revised manuscript critically for intellectual content; approved final version to be published; and agreed to be accountable for all aspects of the work in ensuring that questions related to the accuracy or integrity of any part of the work are appropriately investigated and resolved. HJ-B and NW: contributed to design of the trial and acquisition and interpretation of data; co-led the development of the neuroimaging protocols and training of neuroimaging staff in clinical centres; revised manuscript critically for intellectual content; final approval of version to be published; and agreed to be accountable for all aspects of the work in ensuring that questions related to the accuracy or integrity of any part of the work are appropriately investigated and resolved. NK: contributed to design of the neurophysiology aspects of the trial and acquisition and interpretation of data; assisted with the development of the neurophysiology protocols; co-led training of blinded assessors who conducted the neurophysiology measures; led neurophysiology data collection at one clinical centre and coordinated across centres; revised manuscript critically for intellectual content; final approval of version to be published; and agreed to be accountable for all aspects of the work in ensuring that questions related to the accuracy or integrity of any part of the work are appropriately investigated and resolved. EC: contributed to design of the clinical efficacy aspects of the trial; led provision of one of the experimental therapies at one clinical centre and coordinated adherence to therapy protocol across the centres, contributed to training of research therapists in all centres; contributed to interpretation of data; revised manuscript critically for intellectual content; approved final version to be published; and agreed to be accountable for all aspects of the work in ensuring that questions related to the accuracy or integrity of any part of the work are appropriately investigated and resolved. CW: contributed to design and statistical aspects of the trial; liaised with the CTU regarding analysis of data; contributed to interpretation of data; revised manuscript critically for intellectual content; approved final version to be published; and agreed to be accountable for all aspects of the work in ensuring that questions related to the accuracy or integrity of any part of the work are appropriately investigated and resolved. JR: contributed to design of the trial and interpretation of data; led development of the neurophysiology protocol; co-led training of blinded assessors who conducted the neurophysiology measures; oversaw collection of neurophysiology measures; revised manuscript critically for intellectual content; approved final version to be published; and agreed to be accountable for all aspects of the work in ensuring that questions related to the accuracy or integrity of any part of the work are appropriately investigated and resolved. AW: contributed to design of the trial and acquisition and interpretation of data; revised manuscript critically for intellectual content; approved final version to be published; and agreed to be accountable for all aspects of the work in ensuring that questions related to the accuracy or integrity of any part of the work are appropriately investigated and resolved. MG: contributed to acquisition and interpretation of data; led neurophysiology data collection at one clinical centre and contributed to coordination across centres; revised manuscript critically for intellectual content; approved final version to be published; and agreed to be accountable for all aspects of the work in ensuring that questions related to the accuracy or integrity of any part of the work are appropriately investigated and resolved. GB: led design of the health economics aspect of the trial and analysis and interpretation of health economics data; revised manuscript critically for intellectual content; approved final version to be published; and agreed to be accountable for all aspects of the work in ensuring that questions related to the accuracy or integrity of any part of the work are appropriately investigated and resolved. NL: contributed to acquisition of data and conduction of the trial from position of clinical trial manager, and training workshops for research therapists and blinded assessors; revised manuscript critically for intellectual content; approved final version to be published; and agreed to be accountable for all aspects of the work in ensuring that questions related to the accuracy or integrity of any part of the work are appropriately investigated and resolved. CH: contributed to design of the clinical efficacy aspects of the trial; led provision of one of the experimental therapies at one clinical centre and coordinated adherence to this therapy protocol across the centres, contributed to training of research therapists in all centres; revised manuscript critically for intellectual content; approved final version to be published; and agreed to be accountable for all aspects of the work in ensuring that questions related to the accuracy or integrity of any part of the work are appropriately investigated and resolved. RL: contributed to design of the trial particularly neurological aspects; revised manuscript critically for intellectual content; approved final version to be published; and agreed to be accountable for all aspects of the work in ensuring that questions related to the accuracy or integrity of any part of the work are appropriately investigated and resolved. AD: contributed to analysis and interpretation of health economics data; drafted the health economics sections of the paper; approved final version to be published; and agreed to be accountable for all aspects of the work in ensuring that questions related to the accuracy or integrity of any part of the work are appropriately investigated and resolved. JB: contributed to design of the trial; approved final version to be published; and agreed to be accountable for all aspects of the work in ensuring that questions related to the accuracy or integrity of any part of the work are appropriately investigated and resolved. VP: led design of the trial; responsible for overall conduction of the trial; led acquisition and interpretation of data; contributed to analysis of data; led training workshops for research therapists and blinded assessors; drafted manuscript; revised manuscript critically for intellectual content; approved final version to be published; and agreed to be accountable for all aspects of the work in ensuring that questions related to the accuracy or integrity of any part of the work are appropriately investigated and resolved.

## Disclaimer

The views expressed in this publication are those of the author(s) and not necessarily those of the MRC, NHS, NIHR, or the Department of Health.

## Conflict of Interest Statement

The authors declare that the research was conducted in the absence of any commercial or financial relationships that could be construed as a potential conflict of interest. Author VP is an associate editor of Frontiers in Neurology (Stroke) Journal and was recused from all editorial decisions regarding this article. The reviewer MM declared a past coauthorship with one of the authors JB to the handling editor.

## Footnotes

^1^http://www.controlled-trials.com.

^2^http://www.fmrib.ox.ac.uk/fsl.
